# Molecular Subtypes and Risk Prediction Model Based on Malignant Cell Differentiation Trajectories in Breast Cancer

**DOI:** 10.1111/jcmm.70680

**Published:** 2025-08-08

**Authors:** Penghui Yan, Hanlin Sun, Siqiao Wang, Runzhi Huang, Chaofeng Shi, Qihang Yang, Yibo Qiao, Haonan Wang, Deqian Kong, Jiwen Zhu, Yunqing Yang, Zongqiang Huang

**Affiliations:** ^1^ Department of Orthopedics The First Affiliated Hospital of Zhengzhou University Zhengzhou China; ^2^ Department of Burn Surgery The First Affiliated Hospital of Naval Medical University Shanghai China; ^3^ Research Unit of Key Techniques for Treatment of Burns and Combined Burns and Trauma Injury Chinese Academy of Medical Sciences Shanghai China; ^4^ Tongji University School of Medicine Shanghai China; ^5^ Department of Breast Surgery The First Affiliated Hospital of Zhengzhou University Zhengzhou China

**Keywords:** breast cancer (BRCA), cell fate‐related markers, molecular subtypes, prediction model, single‐cell RNA sequencing (scRNA‐seq), spatial transcriptome

## Abstract

Breast cancer (BRCA) is characterised by complex cellular heterogeneity and differentiation hierarchies, which play a crucial role in bone metastasis and therapeutic resistance. However, existing classification systems remain inadequate in capturing these complexities, limiting their effectiveness in guiding treatment strategies. To address this gap, we integrated single‐cell RNA‐seq profiles, spatial transcriptomes, along with 1097 bulk RNA‐seq profiles of TCGA‐BRCA cohort to dissect the molecular landscape of BRCA. By performing UMAP analysis, we identified nine tumour clusters and three spatially distinct spot types (immune, stromal and malignant spots) and further delineated 11 differentiation states from 2493 malignant spots. Through clustering, monocle 2 pseudo‐time and prognostic analyses, we identified the prognostic BRCA cell fate‐related genes, then constructed a novel BRCA stratification system (four subtypes) with differential prognosis, biological plausibility and clinical significance. Also, least absolute shrinkage and selection operator (LASSO) regression analysis was performed for the BRCA cell fate‐related genes in constructing a prognostic model. The model has modest accuracy and accordance (AUC = 0.708), which could distinguish BRCA patients into high‐risk or low groups. With correlation analysis, regulation networks were constructed for different subtypes based on the key cell fate‐related genes, transcription factors, metastasis‐related pathways, immune components and so on, to investigate the regulatory relationships between primary BRCA and BRCA bone metastasis. Afterwards, we identified the most significant inhibitors (puromycin, MS‐275, megestrol, aesculetin) for bone metastatic BRCA, which might have potential translational significance. In all, we developed a novel molecular stratification system for BRCA based on the cell fate‐related markers of malignant cells, which offered strong translational potential for diagnosis, prognosis and personalised therapeutic interventions.

## 1 Introduction

Breast cancer (BRCA) is the most prevalent malignancy among women and remains a leading cause of cancer‐related mortality worldwide [1]. Breast cancer is a complex disease influenced by multiple risk factors, including genetic predisposition, microenvironmental influences and endocrine‐related factors. Despite advancements in diagnostic techniques and multimodal treatment approaches, the prognosis of BRCA patients remains suboptimal [2, 3].

Notably, approximately 75% of advanced BRCA cases develop bone metastases, which are considered incurable, with a median survival of only 2–3 years postdiagnosis. Current clinical strategies remain ineffective due to the limited understanding of the molecular mechanisms driving BRCA progression and metastasis [4, 5]. While various potential biomarkers have been identified through animal models, our knowledge of the invasion and bone metastatic mechanisms is still superficial. A deeper and more comprehensive understanding of the molecular mechanisms underlying BRCA metastasis is crucial for improving prognosis and developing effective therapeutic interventions.

Traditional studies, including bulk transcriptome sequencing and temporal immunofluorescence staining, have provided insights into the cellular dynamics during BRCA tumorigenesis, invasion and metastasis. However, these methods fail to capture the intricate spatiotemporal heterogeneity of individual cells within the tumour microenvironment. The lack of spatial and temporal information presents a major limitation in existing therapeutic approaches, as tumour cells exhibit heterogeneous responses to invasion and metastasis depending on their anatomical location and time‐dependent interactions. Moreover, the complex interplay between tumour epithelial and mesenchymal cells, as well as ongoing cellular differentiation processes, remains largely unexplored. This gap in knowledge significantly hinders the development of effective therapeutic strategies targeting BRCA metastasis [6].

To overcome these limitations, we conducted a comprehensive transcriptome analysis integrating single‐cell RNA sequencing (scRNA‐seq) and spatial transcriptomics to decode BRCA pathogenesis at a spatiotemporal level. Through UMAP analysis, we identified 9 tumour clusters and 3 distinct spot types (immune, stromal and malignant spots) and further delineated 11 differentiation states from 2493 malignant spots. Then, the prognostic BRCA malignant cell fate‐related gene set was identified at the intersection of statistically significant genes from trajectory analysis and survival analysis. More importantly, we developed a novel BRCA stratification system (Subtype I–IV), in which prognosis worsens progressively, validating through multiomics correlation and survival analysis. Furthermore, we constructed regulatory networks based on key cell fate‐related genes, metastasis‐associated pathways, transcription factors (TFs), immune components and proteins, providing critical insights into potential biomarkers and therapeutic targets for BRCA at both spatial and temporal levels.

## 2 Materials and Methods

### 2.1 Data Collection

The present study was endorsed by the Ethics Committee of the Shanghai Tongji Hospital affiliated to Tongji University. Spatial transcriptome of 4325 qualified single spots was collected from the 10X Visium (https://support.10xgenomics.com/spatial‐gene‐expression/sample‐prep/doc/demonstrated‐protocol‐visium‐spatial‐protocols‐tissue‐preparation‐guide) [7]. The scRNA‐Seq data used in our study were collected from the work of Nguyen et al. [8], who utilised the 10X Genomics Chromium (https://www.10xgenomics.com/instruments/chromium‐x‐series) to sequence a total of 24,646 single cells from reduction mammoplastic specimens from 4 nulliparous patients, at an average read depth of 60,000 reads per cell. Mapped read count data from the four patients were obtained from the GEO (https://www.ncbi.nlm.nih.gov/geo/) database (accession number: GSE113197). Gene expression profiles and clinical information of 1063 primary BRCA samples and 34 bone metastatic BRCA samples were collected from the Cancer Genome Atlas (TCGA) database (https://tcga‐data.nci.nih.gov). TFs were obtained from the Cistrome Cancer database (http://cistrome.org/) [9]. Immunologically related gene expression profiles were downloaded from the ImmPort database (https://www.import.org/) [10]. Hallmark pathways were obtained from the Molecular Signatures Database (MSigDB, Version 7.4) (https://www.gsea‐msigdb.org/gsea/msigdb/index.jsp).

### 2.2 Spatial Transcriptome and scRNA‐Seq Analysis

Sample collection, preparation and spatial transcriptome processing were conducted using 10X Visium [7]. Following the procedures of 10× Genomics Chromium [7], the preprocessing of scRNA‐seq data were conducted. Briefly, samples were embedded into precold optical coherence tomography (OCT) and frozen sections in 10 μm thickness were attached on the Visium slides carefully. To determine the optimal permeability time, the Visium Spatial Tissue Optimization Kit (10X Genomics) was utilised to evaluate the permeability time of the samples, which were 6 min for sham samples and 12 min for BRCA samples. Additionally, the Visium Spatial Gene Expression Slide & Reagent kit (10X Genomics) was utilised to generate sequencing libraries, which were sequenced on a NextSeq (Illumina). After demultiplexing of bcl files, results of sequencing were firstly divided into two pair‐ended reads fastq files, which were trimmed to cut polyA tail sequence and the template switch oligo (TSO) sequence. Subsequently, the clean reads of spatial transcriptome were aligned to the GRCh38 (Version: 100) genome assembly and quantified by the Space Ranger Software (Version 1.0.0) (http://10xgenomics.com/), while clean reads of scRNA‐seq were quantified using the Cell Ranger Software (Version 1.0.0) (http://10xgenomics.com/). To verify the stability of the conclusions obtained from spatial transcriptome analysis, multimodal intersection analysis (MIA) was also performed to assess the similarity between the single cell types in the scRNA‐seq and the single spot regions in the spatial transcriptome [11].

The quantified gene expression matrices were analysed utilising Seurat pipeline (Version: 3.2.2) for further analysis [12]. Spots/cells with more than 100,000 transcripts expression and less than 10% mitochondrial gene mapped were extracted for subsequent analysis while genes which expressed in more than two single spot/cells were included into further analysis. Following the QC processes, all samples were integrated into one Seurat object with the ‘IntegrateData’ function, which was then scaled and standardised using the ‘ScaleData’ function. In addition, the ‘vst’ method was performed to determine the top 2000 variable genes. To reduce dimensionality, principal component analysis (PCA) was initially conducted, and the first 20 PCs were merged as input for Uniform Manifold Approximation and Projection for Dimension Reduction (UMAP) and haematoxylin and eosin (HE) staining slides (resolution = 0.50). Likewise, all control samples were also integrated into one single Seurat object and processed by all aforementioned analysis procedures.

### 2.3 Differential Expression Gene (DEG) Analysis

The ‘FindAllMarkers’ function with ‘wilcox’ method was used to identify scRNA‐seq DEGs between normal control samples and BRCA samples from top 2000 variable genes. Only genes with |log_2_Fold Change (FC)| > 0.5 and false discovery rate (FDR) < 0.05 were defined as DEGs.

### 2.4 Spot/Cell Type Annotation

To accurately identify the specific spot or cell type of each unsupervised cluster, differentially expressed genes (DEGs) within subclusters were used as potential reference markers. These were then integrated with well‐established cell surface biomarkers from CellMarker to achieve a comprehensive and precise annotation of spot and cell types [13]. Given the variable gene expression patterns in BRCA, a specialised cellular annotation approach was applied in this section. First, well‐characterised cell surface biomarkers were used to annotate specific spot/cell types, including CD44, FTL and LGALS1 for stromal spots, ALDH18A1, CD24 and MIR205HG for malignant spots, and HLA‐DRA, HLA‐DRB1 and HSPE1 for immune spots. To further illustrate these annotations, cellular feature plots, violin plots, dot plots and heat maps were generated using the Seurat R package (Version 3.2.2), providing a detailed visualisation of marker gene expression across different spot/cell types [12].

### 2.5 Cellular Communication Analysis

To uncover key cellular communication patterns and ligand–receptor interactions among different spot/cell types in BRCA, we conducted cellular communication analysis using the iTALK R package (Version 0.1.0) (https://github.com/Coolgenome/iTALK) [14]. Furthermore, the normalised expression matrix of these genes was integrated into the iTALK object using the ‘rawParse’ function. Eventually, the top 200 ligand–receptor pairs were illustrated in ligand–receptor plots and iTALK networks. In addition, 50 hallmark signalling pathways were obtained from the MSigDB (Version 7.4) (https://www.gsea‐msigdb.org/gsea/msigdb/index.jsp) [15]. Then, Gene Set Variation Analysis (GSVA) (Version: 1.38.0) was carried out to evaluate the absolute quantification of the hallmark pathway activities in BRCA [16].

### 2.6 Trajectory Analysis

To determine the pseudo‐time of each spot type aforementioned in BRCA, trajectory analysis was conducted using Monocle R package (Version: 2.18.0) [17]. Firstly, malignant spots of BRCA samples were extracted and subjected to Monocle2 in R [18]. Next, the matrix of the top 2000 variable genes, identified using the ‘FindVariableFeatures’ function, was used to order the previously defined spot types and identify differentiation fate‐related genes at various time points. Notably, each differentiation fate‐related gene played a crucial role in directing distinct cell fates. Moreover, alterations in the expression patterns of these differentiation fate‐related genes influenced the cellular composition within the BRCA microenvironment. Ultimately, the pseudo‐time trajectory of each spot type at different time points was determined using the ‘reduceDimension’ function with the DDRTree method, and the results were visualised in trajectory plots.

### 2.7 ConsensusClusterPlus Analysis

To identify unknown possible clusters of DRGs based on intrinsic characteristics, ConsensusClusterPlus analysis was performed using GenePattern software (Version: 2.0) [19, 20]. Additionally, the confidence in the number of clusters and the cluster memberships were assessed on the basis of quantitative and visual ‘stability’ evidences.

By subsampling the proportion of features from cell fate‐related gene clusters, each subsample was then partitioned into up to *k* clusters using the agglomerative hierarchical clustering algorithm. Briefly, this analysis process was repeated nine times. The proportions of clusters in which two cell fate‐related genes were grouped together were defined as pairwise *consensus* values, which were computed and integrated into a consensus matrix (CM) for each *k*. Finally, for each *k*, an agglomerative hierarchical consensus clustering based on the distance of 1 − *consensus* values was completed and divided into *k* clusters, which were defined as consensus clusters.

### 2.8 Principal Component Analysis (PCA) and Clinical Correlation Analysis

The principal component analysis was applied to calculate the PCA BRCA score of the optimal *k* (number of clusters) based on cell fate‐related gene expression patterns [21]. In order to better understand the relationship between PCA BRCA score and other multiomics data including genomics, transcriptomics, proteomics and epigenetics, microsatellite instability (MSI), tumour mutation burden (TMB) and PCA BRCA score were integrated into the correlation analysis. The association between PCA BRCA score and immune cells was evaluated using single‐sample gene set enrichment analysis (ssGSEA) [22]. The ssGSEA score was calculated to quantify the enrichment level of immune signatures in BRCA.

Immunotherapy, especially the immune checkpoint inhibitor therapy, has shown promising results in the treatment of BRCA. To accurately predict the prognosis of BRCA patients and their sensitivity to immunotherapy and to increase our understanding of the clinical individualised medication diagnosis and treatment, data on immunotherapy sensitivity of TCGA‐BRCA patients were obtained from The Cancer Immunome Atlas (TCIA) database (https://tcia.at/) [23]. Subsequently, based on the sensitivity scores of TCGA‐BRCA patients to programmed cell death protein 1 (PD‐L1) and (cytotoxic T lymphocyte antigen‐4) inhibitors in the TCIA database, the differences in sensitivity to immunotherapy between the groups with low‐ and low‐PCA BRCA scores were analysed.

### 2.9 Anti‐BRCA Drug–Response Prediction Based on Consensus Clusters

Individualised treatments require the most effective regimen tailored to each patient, as responses to therapy may vary across different consensus clusters (hypothetical BRCA subtypes). Given its direct impact on patient outcomes, accurately assessing the anti‐BRCA drug response for specific BRCA subtypes is essential for advancing precision medicine. Here, two comprehensive databases containing pharmacologic profiling of anticancer compounds, large‐scale genomic expression profiles and experimentally validated drug response measurements were utilised for performance evaluation. Drug response was assessed using IC50 values (the concentration of a drug required to achieve 50% inhibition in vitro, expressed as the natural log of μM) or experimental activity areas (AAs). To ensure consistency, genomic expression profiles were standardised across BRCA cell lines, allowing for the construction of a similarity matrix to compare these cell lines.

In Cancer Cell Line Encyclopedia (CCLE) database (https://portals.broadinstitute.org/ccle) [24], there are 1036 tumour cell lines with both drug‐response profiles of 24 anticancer drug compounds and genomic expression profiles of 18,900 genes available. We selected 1019 cancer cell lines, including 45 BRCA cell lines, for which both the RNA expression data of 57,820 genes and drug response were available. For BRCA cell lines, their similarity with the hypothetical BRCA subtypes in this study was assessed using the Pearson correlation coefficient (PCC) between the gene expression profiles of cancer cell lines. For Drug Sensitivity in Cancer (GDSC) database (release‐5.0; https://www.cancerrxgene.org) [25], there are 655 tumour cell lines with drug response data, measured using AUC (where higher sensitivity corresponds to lower AUC values) and IC50 (where higher sensitivity corresponds to lower IC50 values), along with expression profiles for 12,072 genes. For this study, we selected 453 drugs and 45 BRCA cell lines, ensuring that both drug response data (IC50 and AUC values) and gene expression profiles were available. The gene expression profiles of these 45 BRCA cell lines were utilised to characterise their molecular features. Next, we constructed both a cell line similarity matrix and a drug similarity matrix, incorporating cell line and drug compound characteristics. The Pearson correlation coefficient (PCC) was then computed to assess the similarity between each BRCA cell line and the four consensus clusters (hypothetical BRCA subtypes) identified in this study. Finally, to evaluate differences in drug response across the four BRCA subtypes, we performed a Kruskal–Wallis test to compare AUC values among subtypes. This analysis allowed us to identify potential BRCA subtype‐specific drugs that may be clinically effective for personalised BRCA therapy. Finally, we performed 10‐fold cross‐validation to assess the performance of the anti‐BRCA drug–response prediction method in the aforementioned data sets, respectively.

### 2.10 Quantification of the Hallmarks of Cancer Gene Sets by GSVA

Activities of the 50 hallmark gene sets obtained from the MSigDB database (Version 7.4) were absolutely quantified in each single spot by GSVA.

### 2.11 Functional Enrichment Analysis

Gene Oncology (GO) and Kyoto Encyclopedia of Genes and Genomes (KEGG) functional enrichment analyses were performed to explore the signalling pathways [26]. Biological processes where DEGs were enriched were identified using the ‘cluster Profiler’ R package [27] with thresholds of *p* < 0.01 and FDR < 0.05. In addition, gene set over‐representation analysis (ORA) was conducted to assess the enrichment of specific gene sets within the tested clusters, providing insights into the biological significance and functional distinctions among them [28]. In this study, 26 BRCA‐related gene sets downloaded from the MSigDB (Version 7.4) were categorised into 8 clusters based on similar functional characteristics, including Cluster 1 to Cluster 8 (C1–C8) and hallmark pathway cluster (H) [15]. Furthermore, ORA was performed to identify the functional enrichment of these gene sets in eight clusters (https://github.com/tomastokar/gsoap).

### 2.12 Construction of a Prognostic Risk‐Related DEMFRG Signature Model

The DEMFRG expression profiles and clinical data were integrated to construct an effective prognostic prediction model for BRCA. RNA‐seq data of 1063 primary BRCA samples and 34 bone metastatic BRCA samples from TCGA database were analysed. BRCA patients with assessable and complete clinical information (*n* = 1092) were then randomised approximately 7:3 to a training set (656 samples) and a testing set (436 samples). Then, the training set was applied to construct a prognostic signature, which was evaluated in the testing set.

The univariate Cox proportional hazards regression analysis was performed to determine those DEMFRGs significantly related to patients' prognosis (*p* < 0.001) in the training set. Furthermore, least absolute shrinkage and selection operator (Lasso) regression analysis was conducted to eliminate those prognostic‐related DEMFRGs that were positively correlated with each other to avoid overfitting. Moreover, to identify independent prognostic factors, the prognostic risk‐related DEMFRGs were subjected to the multivariate Cox proportional hazards regression analysis. Eventually, we extracted 15 prognostic risk‐related DEMFRGs as critical candidates for the construction of the prognostic signature. Importantly, the risk score was utilised as a predictor of prognostic status in the prediction model, and the formula for calculation is as follows [29]:

Risk Score=β1×gene1+β2×gene2+β3×gene3…+βn×genen

Specifically, the order number of the corresponding gene in the prediction model was defined as ‘*n*’, the regression coefficient of a gene was defined as ‘β’, and gene_*n*_ represented the expression level of the nth gene for each BRCA sample, respectively.

### 2.13 Evaluation of the Prognostic DEMFRG Signature in Training Set and Validation in Testing Set

Based on the median risk score as the cut‐off value, BRCA patients in the training set were categorised into low‐risk score group and high‐risk score group. The Kaplan–Meier (K‐M) survival curve was constructed to compare the survival rates of these two groups. Additionally, the receiver operating characteristic curve (ROC) was used to evaluate the sensitivity and specificity of the prediction model, as well as the accuracy at 1, 2 and 3 years using the survival ROC and time‐survival ROC package, respectively.

The BRCA patients were ranked based on the risk score. Furthermore, the number of censored patients and the risk score distribution, as well as the prognosis‐related DEMFGs in low‐ and high‐risk groups were visualised using scatter dot plot, distribution curve and heat map, respectively. Similarly, we assessed the accuracy of the prognostic risk signature with the same methods in the testing set.

### 2.14 Evaluation of Immune Cell Infiltration in BRCA

The format of the gene expression profiles of BRCA patients was adjusted in accordance with the accepted format of CIBERSORT. Furthermore, these data were uploaded to the CIBERSORT web portal (http://cibersort.stanford.edu/). The original CIBERSORT gene signature file LM22 defined 22 immune cell subtypes, which was used to analyse datasets from normal samples and BRCA samples. CIBERSORT *p* value < 0.05 was included.

The differential analysis was performed between the tumour group and the normal control group to analyse the significantly differential expression patterns of multiple immune cell subtypes. Bayesian method and LIMMA package were utilised to construct a linear model [30]. *p* value < 0.05 was set as the cut‐off value. Furthermore, Pearson correlation coefficient was utilised to explore the correlations among these different subtypes of immune cell infiltrates.

### 2.15 Immunotherapeutic Response Prediction

Here, Tumour Immune Dysfunction and Exclusion (TIDE) algorithm was employed to predict clinical responses in the BRCA patients with high or low‐risk score under immunotherapy. As a computational method, TIDE integrated the expression signatures of T‐cell dysfunction and T‐cell exclusion as well as other critical indicators of immunotherapy to model tumour immune evasion [31]. After uploading the transcriptome profiles, TIDE score of patients with BRCA from the TCGA cohort was obtained from the TIDE website (http://tide.dfci.harvard.edu).

### 2.16 GSEA Preparation

To identify functional enrichment by comparing differentially expressed malignant fate‐related genes (DEMFRGs) with predefined gene sets that share pathways, localisation, functions or other characteristics, gene set enrichment analysis (GSEA) was performed using the clusterProfiler R package (version 3.5) [32]. The FC value of gene expression between the normal control group and tumour group was computed, and the gene list was determined based on the change in |log_2_FC|.

### 2.17 Hallmark Pathway Analysis

Hallmark pathway enrichment analysis was performed using the clusterProfiler R package based on the hallmark gene sets obtained from the MSigDB database (version 7.4) (http://www.gsea‐msigdb.org/gsea/msigdb/) [33], where the adjusted *p* value < 0.05 was used as the cut‐off value. Finally, based on the medians of immunocyte infiltration degree and immune‐related pathway activity as the cut‐off values, all BRCA patients were divided into low group and high group, respectively. The K‐M survival analysis was performed to compare the survival rates of the two groups.

### 2.18 Construction of the DEMFRG Regulatory Network and Co‐Expression Pearson Correlation Analysis

For each consensus cluster (BRCA subtype), we constructed and visualised the DEMFRG co‐expression regulatory network to assess the correlations between prognostic‐related DEMFRGs and other key components, including differentially expressed transcription factors (DETFs), immune cells and pathways. To identify potential regulatory relationships, Pearson correlation analysis was performed to determine the upstream DETFs and downstream immune cells/pathways associated with DEMFRGs, based on their correlation coefficients and statistical significance (*p* values).

### 2.19 ATAC‐Seq Validation

Firstly, we downloaded ATAC‐seq data of BRCA samples in the TGCA cohort of chromatin accessibility landscape of primary human cancers (https://gdc.cancer.gov/about‐data/publications/ATAC‐seq‐AWG) [34]. Then, for each BRCA subtype, we explored the chromatin accessibility in the location for the key DEMFRGs in the regulatory network. Specifically, each of the 23 chromosomes (from 1 to X) was divided into 92 evenly spaced segments, and we counted the total number of binding peaks in those segments. Because the lengths of chromosomes were different, the lengths of the segments were different. Further, the total number of binding peaks was normalised by the peak densities (segment lengths), which were utilised to characterise the chromatin accessibility for the corresponding region. To further determine the statistical significance of the identified chromatin accessibility, the genomic locations of DEMFRGs and chromosome numbers in the original input data were permuted randomly, and the peak densities for each of those segments were computed similarly [35]. This process was assessed using 10‐fold cross‐validation.

### 2.20 Quantitative Statistical Analysis

In this study, only two‐tailed *p* value < 0.05 and FDR < 0.05 were considered to be statistically significant. The statistical analysis processes were conducted with R version 4.0.3 software (Institute for Statistics and Mathematics, Vienna, Austria; www.r‐project.org), Python version 3.6 software (https://www.python.org/), and Strawberry Perl version 5.30.0.1 software (https://www.perl.org/). For descriptive statistics, the mean ± standard deviation was used for continuous variables with a normal distribution. Moreover, the median (range) was used for continuous variables with an abnormal distribution.

## 3 Results

### 3.1 Multimodal Spot/Cell Type Annotation

**FIGURE 1:**
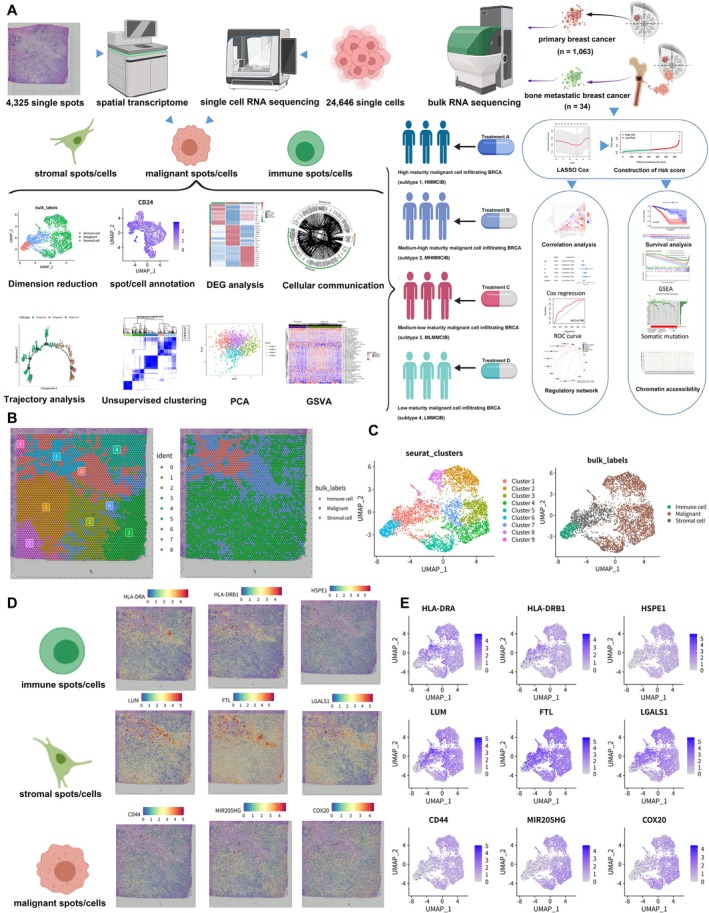
Spatial transcriptome and single‐cell sequencing (scRNA‐seq) and revealed the gene expression landscapes of BRCA cells. (A) Image highlighted vital steps in the workflow. (B) Spatial transcriptome data were utilised to determine the spot subtypes which clearly identified nine clusters. Intraductal carcinoma, invasive ductal carcinoma and normal tissue were shown in the HE staining slides. (C) UMAP plot showed nine cell clusters and three main cell types based on dimension reduction analysis of scRNA‐seq, including immune cells, malignant cells and stromal cells. (D) Spatial transcriptome feature plots and cellular feature plots showed gene expression levels and distribution of representative cell type markers were reported in the CellMarker database for each cell type. (E) Cellular feature plots were utilized to display the expression level of known marker genes of corresponding spot types, which generally defined key genes associated with BRCA.

The schematic diagram and the detailed flowchart of this study are presented in Figure 1A, Figure S1 respectively, illustrating the primary processes of our study. Integrated UMAP analysis based on spatial transcriptome and scRNA‐seq data of unsupervised clustering was performed and clearly identified nine clusters. The dimension reduction analysis based on the unsupervised clustering algorithm was performed to annotate the three main spot types, including immune spots, malignant spots and stromal spots (Figure 1B,C). Spatial feature plots and cellular feature plots showing expression levels and specific distributions of spot type‐specific markers were displayed in Figure 1D. Importantly, it showed that as the cluster of the largest scale of spots, Cluster 1 was composed of stromal spots. In addition, Cluster 6 was composed of immune spots, while the rest of the clusters were mainly composed of malignant spots. Furthermore, cellular feature plots were utilised to display the expression levels of known marker genes of corresponding spot types, which generally defined key genes associated with BRCA (Figure 1E). In total, we identified 2493 malignant spots based on canonical marker genes and clustering, utilised for all further analyses.

### 3.2 Integrated Analysis of Differential Expression Gene (DEG), Cellular Communication and Cell Cycle

DEGs from the top 2000 variable genes were identified in all single spots. Cleveland's dot plot was used to show the expression levels of four key marker genes, which were C3, ARHGDIA, COL1A1 and CDH1 (Figure S2A). In addition, the gene expression levels of nine clusters obtained previously in three types of spots (stromal spots, malignant spots and immune spots) were illustrated by the bar plot.

Multimodal intersection analysis (MIA), iTALK cellular communication analysis and marker genes annotation were performed. For MIA, the hypergeometric distribution was applied to evaluate the statistical significance of the intersection between genes specifically expressed in single cells (scRNA‐seq data) and those identified in spatial transcriptomic spots. This approach ensured a robust assessment of gene overlap, highlighting key molecular interactions across both data modalities. Results of MIA indicated that single cells in the scRNA‐seq and spots in the spatial transcriptome had significantly similar DEGs. The heat map showed the expression levels of the top 10 DEGs in three kinds of spots aforementioned (Figure S2B). Furthermore, the intersected cellular communication network and ligand–receptor plots showed the mechanism of intercellular signal transduction. Importantly, there was significantly strong intercellular communication between malignant spots and stromal spots, which might potentially affect the peritumour microenvironment, a critical factor leading to cancer metastasis (Figure S2C,D). In addition, the cell cycle analysis showed that the majority of these cells were in the G2M and S phases (Figure S2F,G).

### 3.3 Integrated Analysis of scRNA‐Seq, Cellular Communication and Signalling Pathways for Malignant Spots

**FIGURE 2:**
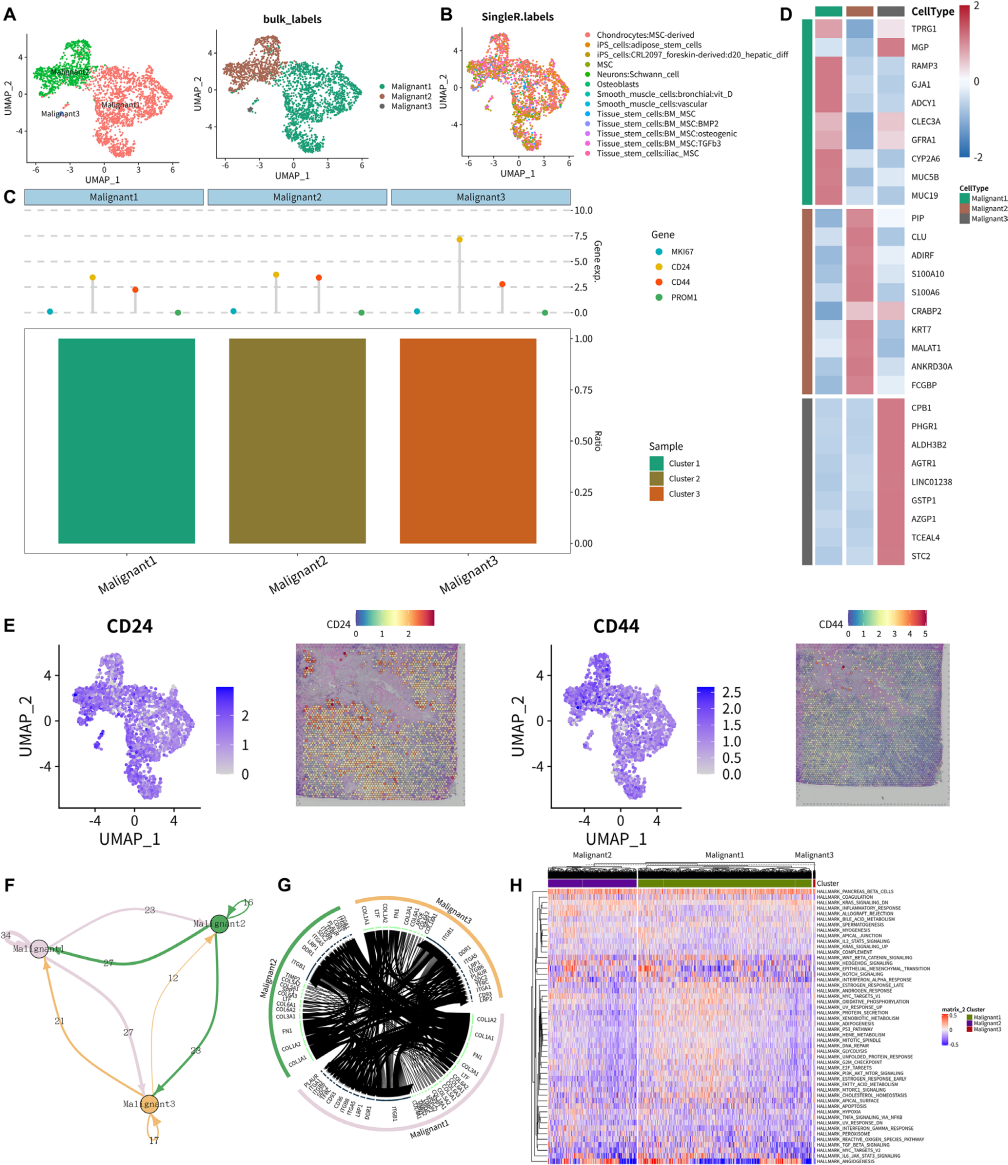
Identification of the BRCA clusters representing malignant cells based on the scRNA‐seq data. (A) UMAP plots of 2493 malignant spots/cells revealing three cell subpopulations. (B) Single R package helped annotate malignant cells with 13 different characteristics. (C) Stacked bar plot with fractions of malignant cells (relative to the total number of each BRCA cluster) shown in the *y*‐axis. Cleveland dot plot showing malignant cell‐specific gene expression levels in each spot/cell subpopulations. Malignant 3 had the highest expression of CD24. (D) The heat map showed the expression levels of the top 10 DEGs of the three different types of malignant cells. (E) CD24 and CD44 were two markers of cancer stem cells. The cellular feature plots and spatial transcriptome feature plots showed gene expression levels and distribution of representative cell type markers (CD24 and CD44) of malignant spots/cells. (F) Network diagram showed the cell–cell communication, each circle indicating one single cell subpopulation. For clarity, thickness of the line represented the strength of the interactions between the two subpopulations. Size of the circle represented the strength of the interactions between the corresponding cell cluster and the other cell clusters. (G) Interaction relationships were noted among Malignant 1, Malignant 2, and Malignant 3, using a circular plot displaying receptor–ligand pairs for indicating all cell subpopulations. (H) Heat map showing the biological features and expression levels of 50 hallmark signalling pathways in malignant cell clusters by Gene Set Variation Analysis (GSVA).

A total of 2493 malignant spots annotated previously were extracted for further analysis of scRNA‐seq, and were first visualised in the UMAP plot (Figure 2A). Then three malignant cell clusters were identified. Similarly, we further annotated these malignant spots using the SingleR package, which were categorised into 13 spot subpopulations (Figure 2B). The major spot subpopulations included 1017 MSC‐derived chondrocytes, 497 tissue stem cell spots, 435 CRL2097 foreskin‐derived iPS cell spots and 400 iPS cell (adipose stem cell) spots. Cleveland's dot plot was constructed to show the expression levels of key marker genes (MKI67, CD24, CD44 and PROM1) in three malignant clusters (Figure 2C). CD24 and CD44 were highly expressed in all malignant clusters, while MKI67 and PROM1 were lowly expressed. Importantly, CD24 has been reported to play an important role in the metastasis of BRCA, the biologic effects of which are related to more rapid cellular growth and strengthened adhesion and invasion ability of human BRCA cells [36]. Furthermore, CD44 is implicated in various biologic features of BRCA cells, such as adhesion, cellular differentiation and metastasis of malignant cells, and cells in BRCA stem cell populations have been confirmed to have a CD44 (+) phenotype [37].

When performing MIA, the hypergeometric distribution was utilised to infer the significance of the intersection of genes specifically expressed in malignant cells in the scRNA‐seq and malignant spots in the spatial transcriptome. Likewise, results of MIA indicated that malignant cells in the scRNA‐seq and malignant spots in the spatial transcriptome had significantly similar DEGs. Afterwards, the heat map showed the expression levels of the top 10 marker DEGs in 3 malignant clusters respectively (Figure 2D). Cellular feature plots and spatial feature plots showing expression level and specific distribution of CD24 and CD44 are displayed in Figure 2E.

Hypothetical ligand–receptor pairs among each spot subpopulation were determined after comparing ligand receptors with these spot‐specific marker genes. Importantly, there was strong intercellular communication between three malignant spot clusters (Figure 2F,G). To further explore the molecular mechanisms underlying malignant cell lineages in BRCA metastasis, biological characteristics and expression levels of 50 cancer‐related hallmark signalling pathways were illustrated in the heat map, which revealed that most of the DEGs were significantly enriched in the following hallmark signalling pathways: hallmark pancreas β cells, hallmark WNT‐β catenin signalling, hallmark epithelial mesenchymal transition, hallmark IL6 JAK STAT3 signalling and hallmark angiogenesis (Figure 2H).

### 3.4 Trajectory Analysis

**FIGURE 3:**
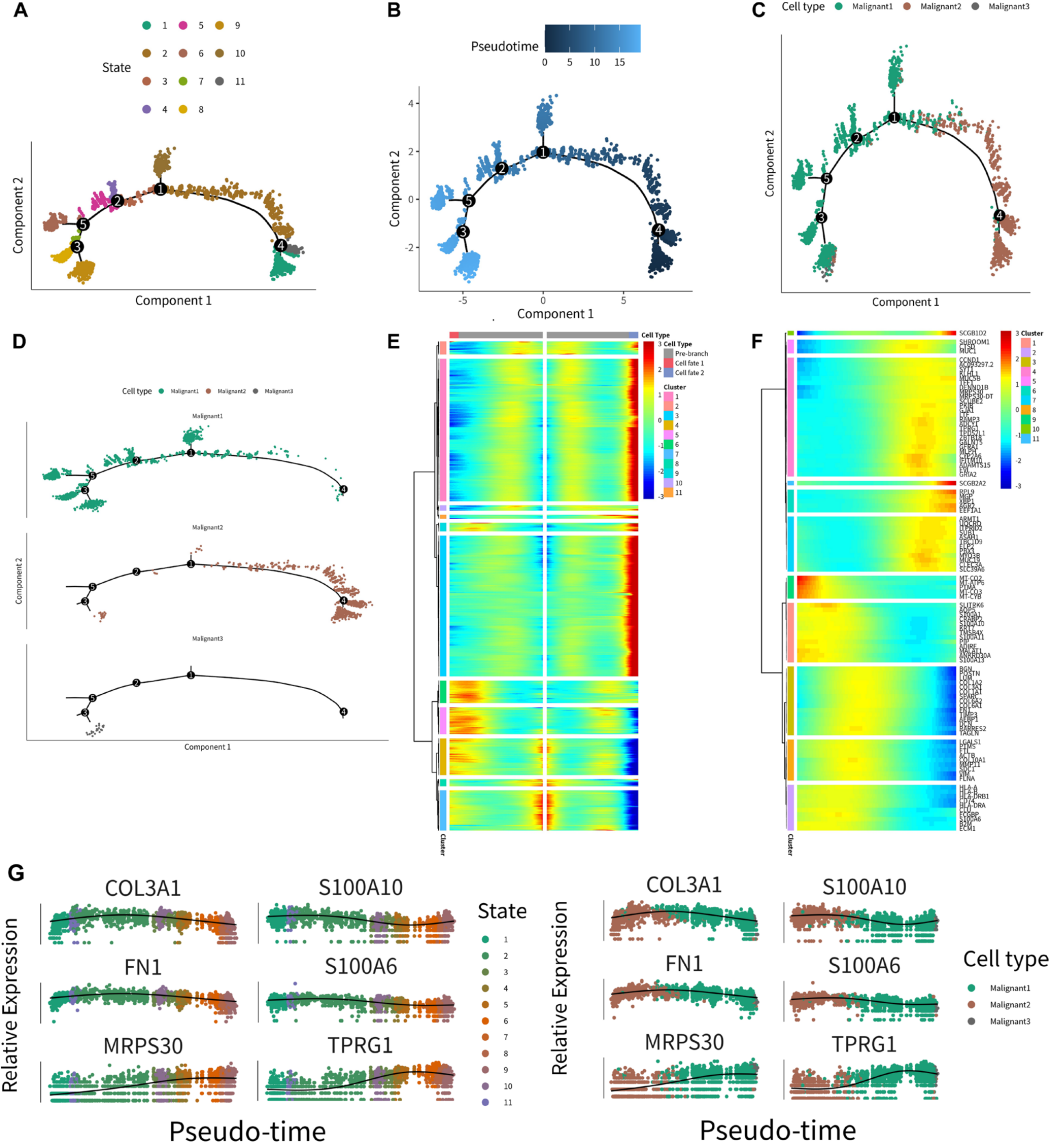
Pseudo‐time analysis revealed malignant cells developmental pathway and fate‐related genes by Monocle 2. (A) Cell trajectory state plot showed developmental trajectory of malignant cells during different pseudo‐time, where 11 cell states were illustrated in different colours and all subpopulations were demonstrated in the trajectory. (B) Pseudo‐time plot showed the simulation of differentiation order of malignant cells. (C) Cell type plot showed all cell clusters along the whole differentiation trajectories. (D) Cell type plot showed each cell cluster on different differentiation trajectories. (E) Heat map displayed alternation of branch specific DEGs expression patterns in branch point 1 in Figure 3C. Unsupervised clustering was conducted to generate the pseudo‐time clusters for fate‐related genes. (F) Heat map represented expression changes of the top 100 representative fate‐related gene differentially expressed across pseudo‐time (*q* < 0.05). (G) We specifically demonstrated the relative expression of the six most significant genes in different pseudo‐time. The results visualised that the expression of many genes varied greatly during different pseudo‐time, indicating their possible regulatory roles.

To simulate gene expression dynamics over the continuous development process during bone metastasis of BRCA, a trajectory analysis based on the transcriptional changes during cell differentiation was performed to reconstruct a cell‐developmental trajectory by Monocle 2. Importantly, malignant spots bifurcated into vastly different gene expression states at each time point, branch point 1–5 separating the cell trajectory into 11 future cellular states (Figure 3A, Figure S3A,B). Secondly, pseudo‐time of all spots was shown in Figure 3B, which elaborated on the differentiation order of these malignant spots. Then, all spot types were illustrated using trajectory plots (Figure 3C,D). In the reconstructed branched tree, starting point 4 can reach different cell fates via various intermediate spot subpopulations. Importantly, Figure 3C showed that spots in Malignant 2 cluster were located at the starting points of the pseudo‐time trajectory in BRCA, while spots in Malignant 3 and Malignant 1 clusters were located at the terminal points.

Key genes possess decisive role in cell fate specification. Therefore, the first bifurcation point (branch point 1) was investigated subsequently, and differential expression analysis was performed to compare two primary branches (cell fate 1 vs. 2). The heat map showed the expression levels of genes in these clusters, which determined the direction of cell differentiation (Figure 3E). Specifically, in Cluster 4–8, high gene expression levels suggested strong cell fate 1 differentiation tendencies. However, in the rest spot clusters, high expression levels suggested strong cell fate 2 differentiation tendencies.

To further explore sequential gene expression dynamics across each branch, DEG analysis, along with the Monocle 2 trajectory analysis, was carried out. Specifically, expression levels of the top 100 state‐related genes in these clusters were shown in the heat map (Figure 3F). Notably, Clusters 1, 2, 3, 8 and 9 were enriched for genes highly expressed early while not sustained across pseudo‐time. By contrast, gene expression levels of Clusters 4, 5, 6, 7, 10 and 11 were highest at late time points. Visualisation of significant differentially expressed genes (COL3A1, FN1, MRPS30, S100A10, S100A6 and TPRG1) based on the results of scRNA‐Seq analysis suggested that these key genes may play central roles in all differentiation stages, considering the continuous peak expression of them tended to be observed throughout the whole pseudo‐time. Furthermore, MRPS30 and TPRG1 were identified to be upregulated in late trajectories, most abundant in spots of Malignant 1 cluster (Figure 3G).

### 3.5 Identification of Prognostic BRCA Cell Fate‐Related Gene Get Associated With Cell Trajectory

To get further insight into the role of genes as cell fate determinants, following the key gene set (507 DEGs, *p* < 0.05) and the ordering gene set (249 genes used for ordering in Monocle 2), we extracted a total of 245 BRCA‐state‐related genes that were differentially expressed across pseudo‐time through the intersection of the aforementioned two gene sets. Furthermore, survival analysis was conducted using the Kaplan–Meier (KM) method and univariate Cox (uniCox) regression model on the basis of gene expression profiles of BRCA RNA‐seq data and clinical information obtained from the TCGA database. In total, 337 KM significant (KM‐sig) DEGs shared 89 genes overlapping with 89 Uni Cox significant (uniCox‐sig) DEGs. Finally, by taking the intersection of these statistically significant results, 43 genes (prognostic BRCA cell fate‐related gene set) were extracted for the subsequent analysis (Figure S4A,B). The KM survival curve of these prognostic BRCA cell fate‐related genes was further constructed, demonstrating the survival probability of patients with BRCA in the TCGA cohort (Figure S4C).

### 3.6 Clustering Analysis Based on ConsensusClusterPlus

**FIGURE 4:**
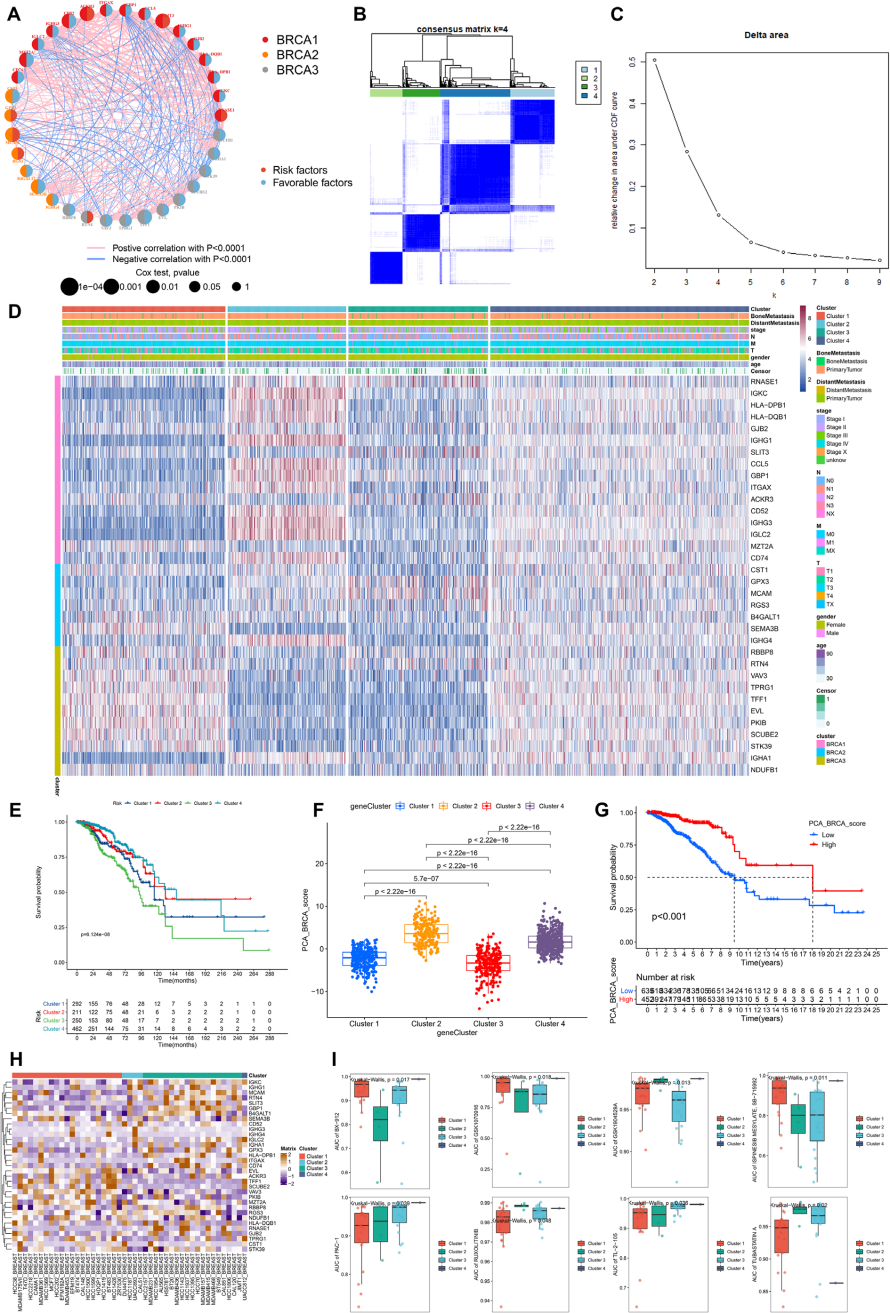
Using Prognostic BRCA cell fate‐related genes for the consensus clustering of BRCA TCGA cohorts. (A) A regulation network for 34 prognostic BRCA cell fate‐related genes. The circles were represented by genes, the left half of colours of which corresponded to malignant cell fates, while the right half of colours depicted the high expression of the gene that was associated with a good (blue) or poor (red) prognosis. Lines indicated statistically significant correlation (*p* < 0.0001). Pink represented the positive correlation and blue showed the negative correlation. Circle size was inversely proportional to the *p* value (Cox test). (B) Identification of four clusters of BRCA bulk RNA‐seq data in TCGA by Consensus clustering analysis, based on 34 prognostic BRCA cell fate‐related genes. The heat map corresponding to the consensus matrix for *k* = 4 was constructed, with the samples represented by columns and rows. Consensus values, reflecting the degree of consensus, ranged from white (0, not clustered together) to blue (1, always clustered together). (C) CDF delta area curve demonstrated that the area under the CDF curve showed no appreciable variation after clustering number *k* = 4. (D) Cluster heat map of the four clusters based on Consensus clustering for prognostic BRCA cell fate‐related gene set. (E) Kaplan–Meier survival analysis showed the differences in the probability of survival in four Consensus clusters. (F) PCA score plot based on the prognostic BRCA cell fate‐related gene indicated that the samples from different Consensus clusters were clearly separated. (G) Kaplan–Meier curves showed significant correlation between PCA BRCA score and survival probability of BRCA patients (*p* < 0.001). Higher PCA BRCA score indicated a better prognosis. (H) Connecting cell lines to malignant cell clusters of BRCA patients. Correlation of co‐expressed malignant cell clusters and BRCA cell lines using the biomarker gene sets. (I) Box plot that integrated gene expression and drug‐response data to connect groups of BRCA patients, cell lines and drugs.

A co‐expressive regulatory network was constructed based on Kaplan–Meier survival curves and Pearson correlation (Figure 4A). In this network, genes determining three directions of cellular differentiation were indicated by labels in different colours. Curve between paired symbols showed positive and negative correlations between these genes, which indicated the regulatory mechanism underlying the clinical prognosis and malignant cell fate based on this specific gene set. All genes were significantly co‐expressed in the malignant region of bone metastatic BRCA during cellular differentiation (*p* < 0.001). In addition, 34 genes were respectively defined as risk factors and favourable factors based on the results of survival analysis. Briefly, each gene was represented by a colour‐coded circle, where red indicated increasing risk and poor prognosis while blue represented decreasing risk and favourable prognosis. The circle size represented *p* value of uniCox regression analysis on overall survival (OS) of BRCA patients, and those with high expression levels of ACKR3, SLIT3 and RNASE1 had the worst prognosis.

Furthermore, based on this network, the aforementioned genes were extracted for further clustering analysis. These genes were categorised into five clusters based on consistency, which were shown in the consensus matrix plot (Figure 4B). In addition, a delta area plot was constructed to show the relative change of the area under the CDF curve when the parameters took different k values (Figure 4C). The heat map showed the expression levels of these genes in various clusters as well as in different genders, bone metastatic/primary tumour groups, distant‐metastatic/primary tumour groups, ages, censors, TNM stages and tumour stages (Figure 4D). Four consensus clusters were dramatically related to the aforementioned clinicopathological characteristics. Notably, strong associations between BRCA cell fate and clustering were determined. Intermediate cell fate and terminal cell fate (BRCA1 and BRCA2) determinant genes were highly expressed in Consensus Cluster 2, while initial cell fate (BRCA3) determinant genes were highly expressed in Consensus Cluster 1. No significant differences in the expression of cell fate‐determinant genes (BRCA1, BRCA2, BRCA3) were observed in Consensus Cluster 3 (low expression) and Consensus Cluster 4 (high expression) (Figure 4D). Importantly, BRCA1 and BRCA2 were highly expressed in the most mature branches of the pseudo‐time trajectory, corresponding to Consensus Cluster 2, which exhibited a higher infiltration of mature BRCA malignant spots. In contrast, BRCA3, predominantly expressed in the least mature branches, was highly enriched in Consensus Cluster 1, indicating a greater presence of immature, cancer stem‐like malignant spots. Meanwhile, Consensus Cluster 3 showed low expression of nearly all cell fate‐related genes, suggesting a population of malignant spots with multilineage differentiation potential, similar to cancer stem cells, which drive tumour growth and heterogeneity [38]. In Consensus Cluster 4, nearly all cell fate‐related genes, particularly terminal differentiation‐related genes (BRCA3), were highly expressed. This suggests that tumours in these patients were the most well‐differentiated and heterogeneous, comprising multiple phenotypically distinct malignant subpopulations. Kaplan–Meier (KM) survival curves (Figure 4E) demonstrated a significant correlation between clustering and BRCA prognosis (*p* < 0.001). Consensus Cluster 3, characterised by poorly differentiated malignant spots, exhibited the worst prognosis, while Cluster 4, with well‐differentiated malignant spots, had the best prognosis. Moreover, Cluster 2 had a better prognosis than Cluster 3, reinforcing the notion that tumours with more immature malignant spots are associated with poorer survival outcomes. To further assess the discriminatory power of prognostic BRCA cell fate‐related genes, we performed principal component analysis (PCA). The box plot revealed significant differences in PCA BRCA scores among the four clusters (*p* < 0.001), with Cluster 3 displaying the lowest PCA score and the worst prognosis, consistent with previous results (Figure 4F). Additionally, KM survival analysis confirmed that higher PCA BRCA scores correlated with worse prognosis (*p* < 0.001) (Figure 4G). The PCA scatter plot (Figure S5A) highlighted distinct sample distributions across clusters, while the Sankey diagram (Figure S5B) illustrated the proportions of high‐ and low‐scoring components in each cluster and their relationship with prognosis.

Eventually, we labelled four novel molecular subtypes according to the corresponding mature degree of malignant spots and markers, the expression levels of which were significantly higher, including Subtype I (corresponding to Consensus Cluster 4): high maturity malignant cell infiltrating BRCA (HMMCIB), Slit Guidance Ligand 3 (SLIT3) (marker); Subtype II (corresponding to Consensus Cluster 2): medium‐high maturity malignant cell infiltrating BRCA (MHMMCIB), integrin Subunit Alpha X (ITGAX) (marker gene); Subtype III (corresponding to Consensus Cluster 1): medium‐low maturity malignant cell infiltrating BRCA (MLMMCIB), cAMP‐dependent protein kinase inhibitor‐beta (PKIB) (marker gene); Subtype IV (corresponding to Consensus Cluster 3): low maturity malignant cell infiltrating BRCA (LMMCIB), melanoma cell adhesion molecule (MCAM, marker gene), and the prognosis became worse in turn. As expected, in the box plot, the expression level of these genes among different clusters showed a significant difference (Figure S5C), providing a molecular signature that enables precise patient stratification. By assessing the expression levels of multiple key cell fate‐related genes, we can determine the BRCA subtype of different patients.

### 3.7 Connecting Consensus Clusters to Drug Responses of BRCA Cell Lines

Genome‐wide expression profiles from consensus clusters and cell lines in BRCA, together with drug‐response data of these cell lines, open prospects for integrative analyses which may lead to better personalised treatments. Drug responses and RNA expression profiles obtained from the BRCA cell lines were utilised to generate tripartite networks connecting consensus clusters of BRCA patients to cell lines and cell lines to drug compounds, to connect drug compounds to consensus clusters.

To evaluate similarity in RNA expression profiles between the consensus clusters (BRCA subtypes) and cell lines, here we correlated the RNA expression of the cell lines with those of each consensus cluster utilising the BRCA cell fate‐related gene set (Figure 4H). Clustering the correlations between four consensus clusters and BRCA cell lines showed subtype‐specific expression similarity, which enabled matching of BRCA subtypes to various cell lines. For the RNA expression analysis, we identified that Consensus Cluster 1 had similar expression profiles to BRCA cell lines including HCC38, MDAMB175VII, T47D, HCC2218, CAMA1, MDAMB361, HCC1569, MCF7, HCC202, EFM192A, MDAMB453, EFM19, BT474, CAL148, HCC1500, HCC1599, HDQP1, HCC1419, BT483, HCC1428 and ZR7530. Furthermore, strong similarity of Consensus Cluster 2 to DU4475, HCC1187, UACC893 and CAL51 BRCA cell lines, indicating that these cell lines and Consensus Cluster 2 may be similar at the transcriptome level. Of note, we also observed that Consensus Cluster 4 was more similar to the UACC812 BRCA cell line relative to the other cell lines. The higher correlation between Consensus Cluster 3 and the rest of the BRCA cell lines was identified. Once we have determined the multiple consensus clusters and cell lines, we could match them and connect them to the drug responses which showed the most potency in cellular growth suppression and apoptosis for these cell lines.

Next, we analysed the drug‐response data for the 45 BRCA cell lines treated with 453 drugs from the GDSC database. Response data were quantified as the AUC values, which were converted into sensitivity measures; this meant that higher AUC values corresponded to higher sensitivity of a BRCA cell line/consensus cluster to a drug. Eventually, to bring integrated and condensed insights into the associations between the independent data types, we created tripartite box plots which capture the connections between BRCA cell fate‐related RNA expression signature from the cell lines and consensus clusters with drug‐response profiles for the 45 cell lines treated with 453 drugs by Kruskal–Wallis test, illuminating the indirect correlations between consensus clusters and drugs. The most effective drugs for HMMCIB (Subtype I, Cluster 4) included BX‐912, GSK1904529A, ISPINESIB MESYLATE, SB‐715992 and PAC‐1; the most effective drugs for MHMMCIB (Subtype II, Cluster 2) included RUXOLITINIB and TUBASTATIN A; the most effective drugs for MLMMCIB (Subtype III, Cluster 1) included BX‐912, GSK1904529A, ISPINESIB MESYLATE and SB‐715992; the most effective drugs for LMMCIB (Subtype IV, Cluster 3) included PCA‐1, TL‐2‐105 and TUBASTSTIN A (Figure 4I). These findings indicate that each BRCA subtype exhibits distinct drug sensitivities, with specific inhibitors demonstrating the strongest suppression effects on their corresponding clusters, as identified through CCLE and GDSC database analysis.

### 3.8 Immune Infiltrating Characteristics Associated With BRCA Molecular Subtypes

The interaction between immune systems and cancers has emerged as an important aspect of cancer biology and is strongly related to the host's ability to suppress malignant cell growth and to respond to immunotherapies. Hence, incorporating more precise immune‐related information in descriptive cancer‐subtyping research or in prospective clinical trials is of vital importance.

Statistically significant differences in immune infiltration degree were identified among multiple immune‐related cells (Figure S6A). Importantly, almost all immune cells including activated B cell (plasma cell), activated CD4 T cell, and activated CD8 T cell presented a significantly higher degree of infiltration in Cluster 2 (MHMMCIB), whereas the infiltration degree of immune cells was significantly lower in Cluster 1 (MLMMCIB). Likewise, the infiltration degree of almost all immune cells was significantly lower in Cluster 3 (LMMCIB) than in Cluster 4 (HMMCIB). Hence, we can conclude that the more immature the malignant cells, the less infiltration the immune cells, the worse the BRCA patients' prognosis might be.

### 3.9 Correlation Analysis of PCA BRCA Score in Multiomics Dimension

The integrated analysis of spatial transcriptome and single‐cell multiomics was performed so as to unravel the mystery of cellular complexity in BRCA malignant spots. The genomic data of the BRCA patients from the TCGA database were merged in multiomics analyses, determining the association between multi‐dimensional omics signatures and PCA BRCA score as well as consistent clustering.

### 3.10 Immune Infiltration Characteristics

Furthermore, the expression level of PD‐L1 in the high‐PCA BRCA score group was significantly higher than that in the low‐PCA BRCA score group (Figure S6B). Importantly, a higher PD‐L1 expression level indicated better efficacy of PD‐L1 monoclonal antibody immunotherapy. Therefore, the PCA BRCA score may be correlated with the therapeutic response to immunotherapy, and that is, patients with a high‐PCA BRCA score may have favourable outcomes when receiving PD‐L1 targeted immunotherapy. Moreover, the co‐expression heat map showed that the PCA BRCA score positively correlated with the activities of immune‐related cells, which were closely related to the inhibition and removal of malignant cells (Figure S6C).

### 3.11 Role of the PCA BRCA Score in Predicting the Benefit of Immunotherapy

Based on the results above, the differences in efficacy between CTLA4 inhibitor and PD‐1 inhibitor in patients with different PCA BRCA score groups were analysed according to the immunotherapy sensitivity data from the TCIA database. The results indicated that patients in the high‐PCA BRCA score group, previously associated with activities of immune‐related cells, were more sensitive to PD‐1 inhibitors (*p* < 0.001, Figure S6D) as well as CTLA4 inhibitors in combination with PD‐1 inhibitors (*p* < 0.001, Figure S6E). For the group with low sensitivity to immunotherapy, we did not utilise these inhibitors (*p* < 0.001, Figure S6F) or CTLA4 inhibitors only (*p* < 0.001, Figure S6G), which could have more clinical efficiency. These results indicated that PCA BRCA score may be related to immunotherapeutic responses, which may have implications for the selection of immunosuppressive agents in BRCA therapy.

### 3.12 Genome Characteristics

Waterfall plots showed the mutation probabilities in high‐PCA BRCA score group (Figure S7A) and low‐PCA BRCA score group (Figure S7B). Compared with low‐PCA BRCA score group, those with high‐PCA score exhibited relatively higher gene mutation probabilities, which indicated that the genomes of these patients were more unstable. In addition, pathogenic variants frequency of several genes was relatively higher in the high or low score group. Significantly higher incidence of pathogenic mutations in PIK3CA (40% vs. 28%) and CDH1 (18% vs. 7%), indicating a better prognosis, was identified in high‐PCA BRCA score group, which was consistent with the conclusion draw from the above survival analysis that PCA BRCA score could help classify patients into good and poor prognosis groups. Importantly, various previous studies found that high frequency mutations of PIK3CA may exhibit positive impact in hormone receptor (HR)‐positive BRCA [39, 40]. Besides, pathogenic germline mutations of CDH1 were related to lobular BRCA in hereditary lobular breast cancer (HLBC) syndrome [41]. Then, tumour burden mutation (TMB) was assessed among the samples based on the mutation data, and all BRCA patients were categorised into TMB‐high and TMB‐low subpopulations. The KM survival plots showed the long period prognosis was significantly better in patients with high TMB and high‐PCA BRCA score than those with low TMB and low‐PCA BRCA score (*p* < 0.001) (Figure S7C). As a significant parameter for cancer heterogeneity, copy number variation (CNV) was examined for 28 malignant cell fate‐related genes in BRCA samples derived from TCGA. The dot plot was used to show CNV frequencies of 28 genes obtained, and high mutation rates contributed to a poor prognosis. In addition, the circos plot was constructed to show CNV frequencies of these key genes in specific chromosome locations. Frequent losses in CD52, MCAM, SEMA3B, ACKR3, GBP1, VAV3, NDUFB1, SCUBE2 and other genes, involving genomic regions spanning 1, 2, 3, 5, 6, 10, 11 and 14 chromosomes (Figure S7D,E).

### 3.13 Identification of Differentially Expressed Genes (DEGs), Transcription Factors (DETFs) and Signalling Pathways

Based on the threshold, a total of 1784 genes were identified as differentially expressed genes (DEGs) between 1063 primary tumour samples and 34 bone metastatic samples, which were shown in the heat map (Figure S8A) and volcano plot (Figure S8B). Importantly, 28 prognostic BRCA cell fate‐related genes were also differentially expressed between primary BRCA and bone metastatic BRCA samples, which were extracted for further analysis (Figure S8C,D). Thirty‐seven transcription factors (TFs) were defined as differentially expressed TFs (DETFs) between primary tumour and bone metastasis samples based on the threshold, which were shown in the heat map (Figure S8E) and volcano plot (Figure S8F).

Expression levels of 50 cancer‐related hallmark signalling pathways in primary tumour and bone metastasis samples were illustrated in the heat map (|log_2_FC| > 0.1, *p* < 0.05) (Figure S8G). Furthermore, the bar plot showed the *t* value of these hallmark pathways, and hallmark pathways with the absolute value of *t* greater than 1 were extracted for subsequent analysis (Figure S8H).

### 3.14 Establishment of a Prognostic Risk‐Related DEMFRG Signature Model

**FIGURE 5:**
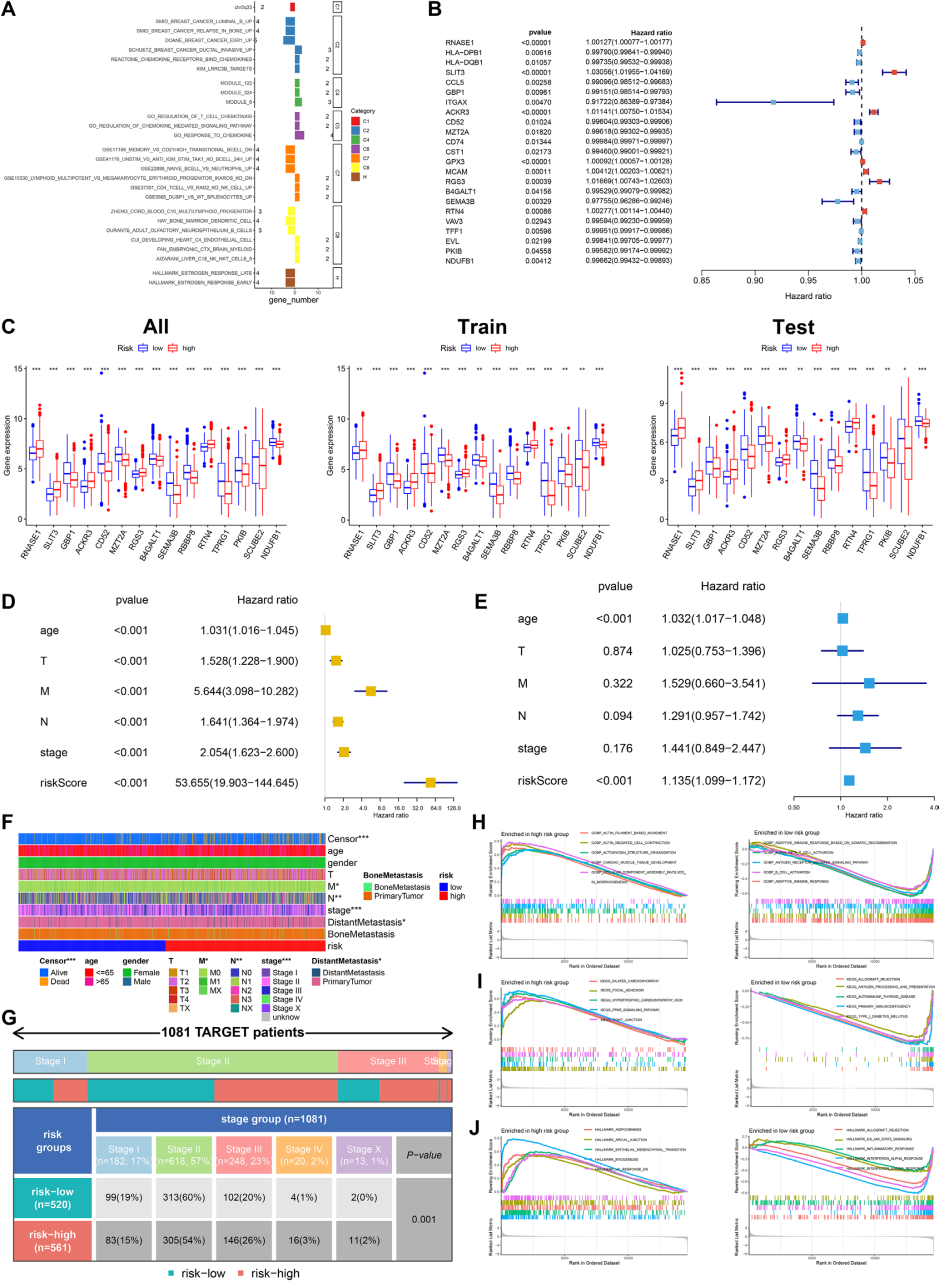
Construction of the prognosis prediction model and regulatory network based on prognostic BRCA cell fate‐related genes. (A) Over‐representation analysis of differentially expressed fate‐related genes in pathways and GO terms was implemented by comparing primary tumour samples to metastatic BRCA. (B) Box plots showed significantly differential expression levels of 15 prognostic malignant cell fate‐related genes between low‐ and high‐risk groups in all sets, train set and test set, respectively. (C) Forrest plot of univariate Cox regression analysis for six clinical parameters affecting prognosis in BRCA, which indicated that risk score was an independent risk factor. (D) Forest plot of univariate Cox regression analysis for six clinical parameters affecting prognosis in BRCA, preliminarily proving that risk score as a significant risk factor. (E) Forest plot of multivariate Cox regression analysis for six clinical parameters affecting prognosis in BRCA, which indicated that risk score was an independent risk factor. (F) Heat map demonstrated the strong clinical relevance of risk score. (G) A large BRCA cohort from the Therapeutically Applicable Research to Generate Effective Treatments (TARGET) database was used to verify the clinical significance of the risk stratification system. (H) GSEA‐based GO analysis. (I) GSEA‐based KEGG analysis. (J) GSEA‐based Hallmark pathway analysis.

In order to further explore the role of genes that were closely related to tumour differentiation, differential expression analysis was performed among the gene clusters we obtained previously. In total, 28 prognostic BRCA cell fate‐related genes were identified to be expressed differently between primary tumour samples and metastasis samples. Differentially expressed prognostic BRCA cell fate‐related genes were mainly enriched in several gene sets of immune response and breast cancer progression (Figure 5A).

Firstly, univariate Cox proportional hazards regression analysis was conducted based on gene expression of 656 BRCA patients in the training set to evaluate the prognostic correlations between gene expression profiles and OS. We observed that the expressions of 23 DEMFRGs were significantly related to the prognosis of BRCA patients (*p* < 0.05) (Figure 5B). Lasso regression analysis and multivariate Cox proportional hazards regression analysis were performed based on the 23 prognostic‐related DEMFRGs, and regression coefficients of them were computed. The coefficient of each DEMFRG was displayed in Figure S9A. Importantly, when it included 15 DEMFRGs, the model achieved the best performance (Figure S9B). Eventually, as illustrated in Figure 5C, 15 DEMFRGs were extracted to construct the prognostic risk‐related DEMFRG signature model using the formula as follows: risk score = (0.1134) * RNASE1 + (0.1126) * SLIT3 + (−0.2472) * GBP1 + (0.0747) * ACKR3 + (−0.0669) * CD52 + (−0.0695) * MZT2A + (0.0646) * RGS3 + (−0.1491) * B4GALT1 + (−0.1600) * SEMA3B + (−0.0620) * RBBP8 + (0.2354) * RTN4 + (−0.0776) * TPRG1 + (0.0440) * PKIB + (−0.0248) * SCUBE2 + (−0.2030) * NDUFB1.

### 3.15 Evaluation of the Prognostic Signature in Training Set and Validation in Testing Set

To verify the reliability of this prognostic risk‐related DEMFRG signature model, the K‐M survival curves were constructed in all sets, training set and testing set. We found that the OS of BRCA patients with low‐risk scores was significantly better than those with high‐risk scores in all sets, training set and testing group (Figure S9C) (all *p* < 0.001). The heat map demonstrated that BRCA patients with high‐risk scores expressed higher levels of risk factors, while BRCA patients with low‐risk scores expressed higher levels of protective factors, indicating there were significant differences between the 15 prognostic‐related DEMFRGs in low‐ and high‐risk score BRCA patients in all sets, training set and testing set (Figure S9D). ACKR3, PKIB, RGS3, RNASE1, RTN4 and SLIT3 were risk factors that were significantly upregulated in the high‐risk score BRCA group, whereas B4GALT1, CD52, GBP1, MZT2A, NDUFB1, RBBP8, SCUBE2, SEMA3B and TPRG1 were protective factors, which were significantly downregulated in the high‐risk score BRCA group (Figure 5C). The scatter dot plots showed that high‐risk BRCA patients had worse survival outcomes than those with low‐risk scores based on the prognostic‐related DEMFRG signature in all sets, training set and testing set (Figure S9E). Significantly different risk score distribution was illustrated between low‐ and high‐risk BRCA patients in all sets, training set and testing set (Figure S9F).

### 3.16 Clinical Significance of the Prognostic Risk‐Related DEMFRG Signature Model

ROC curves indicated that the AUC values for the prognostic risk‐related signature model were 0.708 in all set, 0.717 in the training group and 0.703 in the testing group (Figure S9G). AUC values corresponding to 1‐, 2‐ and 3‐year OS were 0.681, 0.676 and 0.731 in all set. These results indicate that the risk score model exhibits robust prognostic performance across different datasets, effectively stratifying patients based on survival probability and demonstrating its potential clinical utility in improving prognosis prediction and guiding risk‐based therapeutic strategies. We integrated the risk score from the prognostic risk‐related DEMFRG signature and clinicopathological characteristics, including age, TNM stage and AJCC stage. Subsequently, univariate and multivariate Cox regression showed that the risk score was an independent prognostic factor in the univariate (HR = 53.655, 95% CI (19.903–144.645), *p* < 0.001, Figure 5D) and multivariate (HR = 1.135, 95% CI (1.099–1.172), *p* < 0.001, Figure 5E) Cox regression models.

Furthermore, we identified that patients with higher AJCC stages, M stage and N stage had higher risk score than those with lower AJCC stages, M stage and N stage (*p* < 0.05). Additionally, risk score was higher in dead patients and patients with distant metastasis than in alive patients and those without metastasis (*p* < 0.05), whereas there was no significant difference in a different age, gender, T stage and bone metastasis (*p* > 0.05) (Figure 5F). The chi‐square test was performed based on gene expression profiles and clinical information of 1081 BRCA patients obtained from the Therapeutically Applicable Research to Generate Effective Treatments (TARGET) database (https://ocg.cancer.gov/programs/target) [42], which showed the AJCC stages in low‐ and high‐risk score groups had significant difference (*p* = 0.001, Figure 5G). It also suggested the prognostic risk‐related DEMFRG risk score model was correlated with clinicopathological characteristics, which could be independently utilised in predicting clinical outcomes of BRCA patients.

### 3.17 Correlation Analysis of Immune Cells and Immune Gene Sets

A diverse clinical prognosis may occur in patients with the same histological tumour type due to different immune cell infiltration degrees. Therefore, we followed up to explore the landscape of BRCA immune microenvironment using the CIBERSORT algorithm. The bar chart showed the percentages of 22 kinds of immune cells in primary tumour and metastasis samples (Figure S10A). The proportion of macrophages was relatively higher in metastasis samples than that in primary tumour samples. The heat map was used to quantify the correlation among 22 immune cells based on CIBERSORT (Figure S10B). Results of nonparametric tests demonstrated that infiltration degrees of B cells naïve, T cells CD4 naïve, T cells CD4 memory resting, T cells follicular helper, NK cells activated, dendritic cells resting and mast cells resting were lower in bone metastatic tumour samples than in primary tumour samples, while infiltrates of macrophages M0, NK cells resting and neutrophils were relatively higher in bone metastatic tumour samples (***p < 0.001, ** *p* < 0.01, *p < 0.05) (Figure S10C). Furthermore, ssGSEA was conducted to quantify the activities of 23 immune cells between primary tumour samples and metastasis samples, which were shown in the heat map (Figure S10D). The cell activity of macrophage was higher in metastasis samples than that in primary tumour samples, according well with the results by CIBERSORT. Moreover, BRCA patients with high infiltration degree of CCR, macrophages M1, mast cells resting, plasma cells, T cells CD8, T cells follicular helper and T cells regulatory (Tregs) exhibited a better prognosis, whereas those with high infiltration degree of macrophages M2 showed a poorer prognosis (all *p* < 0.05, Figure S10E).

### 3.18 GSEA Analysis and GO Functional Enrichment Analysis

Based on the GO biological processes, the top five most significantly enriched GO terms were illustrated in Figure 5G, where lines represented enrichment profiles. In high‐risk BRCA, prognostic DEMFRGs in GO BP terms were primarily related to ‘GOBP actin filament‐based movement’, ‘GOBP actin‐mediated cell contraction’, ‘GOBP actomyosin structure organization’, ‘GOBP cardiac muscle tissue development’ and ‘GOBP cellular component assembly involved in morphogenesis’. A total of 77 genes were implicated in ‘GOBP cellular component assembly involved in morphogenesis’ with the highest enrichment level (enrichment score (ES) = 0.784), which was very significant in high‐risk BRCA.

In low‐risk BRCA, prognostic DEMFRGs were significantly enriched in ‘GOBP adaptive immune response’, ‘GOBP adaptive immune response based on somatic recombination’, ‘GOBP alpha beta T cell activation’, ‘GOBP antigen receptor‐mediated signaling pathway’ and ‘GOBP B cell activation’. A total of 127 genes were implicated in ‘GOBP alpha beta T cell activation’ with the highest enrichment level (ES = −0.643), which demonstrated immune responses were obviously suppressed in low‐risk BRCA (Figure 5H).

### 3.19 KEGG Analysis and Hallmark Pathway Analysis

A total of 10 prominent KEGG pathways were identified. In the activated pathways, 60 genes participated in the KEGG PPAR signalling pathway, which were concentrated at the front of the sequence with the highest enrichment level (ES = 0.720). The core genes in this pathway were upregulated in high‐risk BRCA. In the suppressed pathways, only 30 genes participated in the KEGG primary immunodeficiency, which were concentrated at the back of the sequence with the highest enrichment level (ES = −0.856). The core genes in this pathway were downregulated in low‐risk BRCA (Figure 5I).

A total of 10 key hallmark pathways including activated and suppressed pathways were selected. In the activated pathways, 185 genes participated in the hallmark myogenesis pathway, which were concentrated at the front of the sequence with the highest enrichment level (ES = 0.790), and core genes in this pathway were upregulated in high‐risk BRCA. In the suppressed pathways, 94 genes were involved in the hallmark interferon alpha response, which were concentrated at the back of the sequence with the highest enrichment level (ES = −0.806). The core genes in this pathway were downregulated in low‐risk BRCA (Figure 5J).

### 3.20 Construction of the Regulatory Network

**FIGURE 6:**
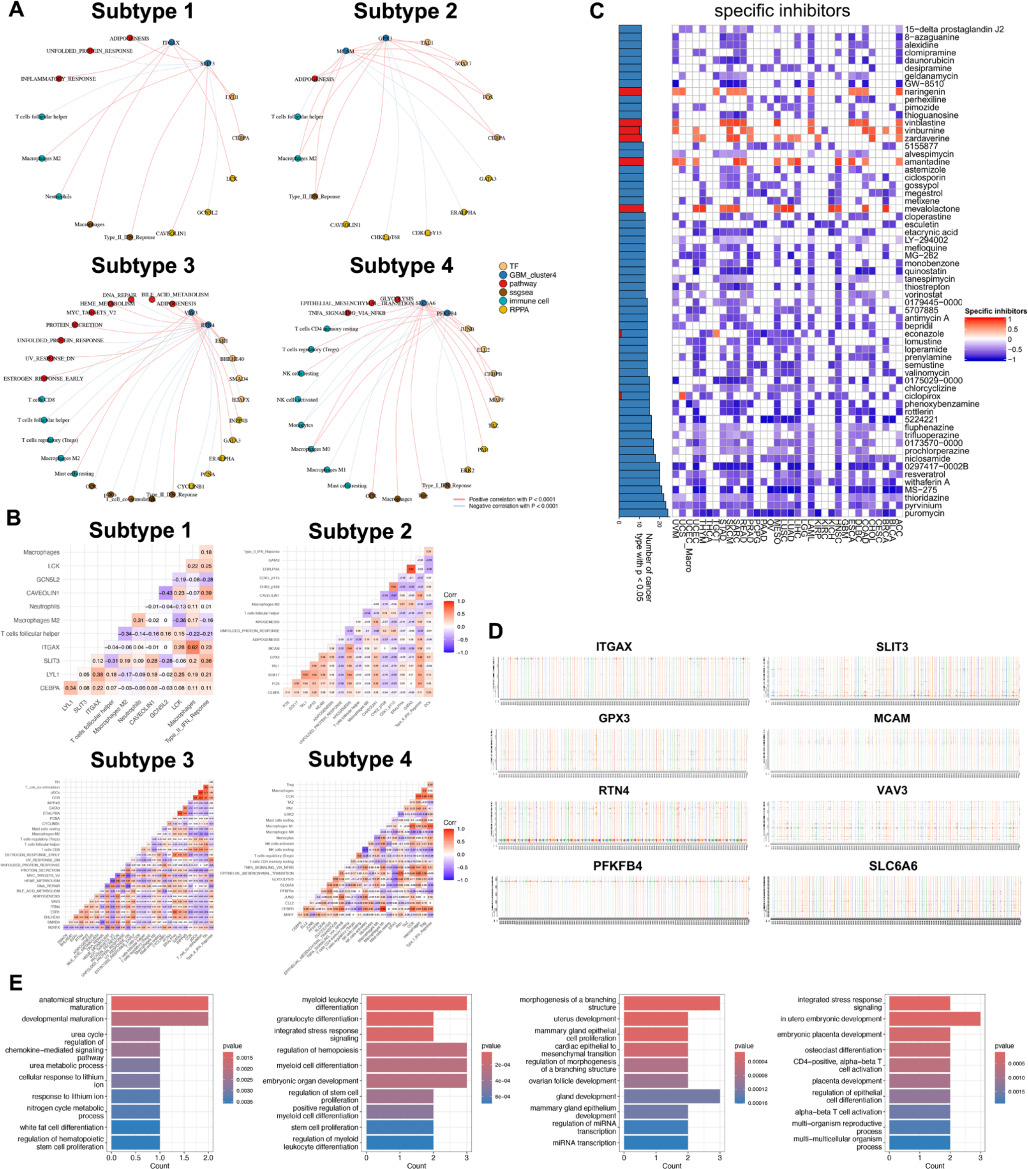
Correlation analysis of regulation network and the identification of potential cell fate‐related gene targeting inhibitors. (A) BRCA bone metastasis regulatory networks along the differentiation trajectories showed tight gene linkage of cooperative expression regulation. Blue circles indicated key differentially expressed BRCA cell fate‐related genes (BRCA cluster 1–4); yellow circles indicated transcription factors (TFs); red circles indicated pathways; brown circles indicated metastasis‐related immune gene set obtained by ssGSEA (ssgsea); dark yellow circles indicated metastasis‐related protein (RPPA); green circles indicated immune cells. (B) Co‐expression heat map showed the co‐expression patterns and correlation among all components. (C) Heat map showed a set of specific inhibitors based on key malignant cell fate‐related genes by CMap analysis. (D) Identification of ATAC‐seq track signals at the indicated multiple loci containing key malignant cell fate‐related genes (ITGAX, SLIT3, GPX3, MCAM, RTN4, VAV3, PFKFB4, SLC6A6), and multiple open chromatin peak profiles represented the chromatin regions demonstrating altered accessibility. (E) Bar plots showed the functional enrichment of key malignant cell fate‐related genes in each regulatory network.

Considering the findings discussed above, we aimed to integrate the prognostic DEMFRG set and its regulators into a broader framework of BRCA bone metastasis. To achieve this, we incorporated upstream regulators (bone metastasis‐related transcription factors, DETFs), midstream factors (key DEMFRGs) and downstream effectors (bone metastasis‐related signalling pathways). Additionally, we analysed bone metastasis‐related immune components, including ssGSEA‐derived immune gene sets, infiltrating immune cells and RPPA‐based protein expression profiles, providing a comprehensive view of the molecular landscape underlying BRCA bone metastasis. The regulatory networks of each subtype were visualised (Figure 6A).

To quantify the interaction coefficients of key biomarkers in each BRCA subtype (DETFs, DEMFRGs, hallmark signalling pathways, RPPA protein chips, immune gene sets and immune cells), correlation analysis was performed at the transcriptional level, which was shown in the heat maps (Figure 6B). Specifically, in Malignant 1, DEMFRG (ITGAX) exhibited a significant co‐expression pattern with DETF lymphoblastic leukaemia derived sequence 1 (LYL1) (*R* = 0.36, *p* < 0.001), inflammatory response pathway (*R* = 0.58, *p* < 0.001), macrophages (*R* = 0.62, *p* < 0.001), RPPA LCK (*R* = 0.28, *p* < 0.001); in Malignant 2, DEMFRG (MCAM) showed a significant co‐expression pattern with DETF (SOX17) (*R* = 0.34, *p* < 0.001), myogenesis pathway (*R* = 0.48, *p* < 0.001), Type II IFN response (*R* = 0.28, *p* < 0.001), RPPA GATA3 (*R* = −0.26, *p* < 0.001). In Malignant 3, DEMFRG (VAV3) showed a significant co‐expression pattern with DETF (BHLHE40) (*R* = 0.48, *p* < 0.001), oestrogen response early pathway (*R* = 0.39, *p* < 0.001), mast cells resting (*R* = 0.22, *p* < 0.001), Type II IFN response (*R* = 0.08, *p* < 0.001) and RPPA GATA3 (*R* = 0.35, *p* < 0.001); DEMFRG (RTN4) showed a significant co‐expression pattern with DETF (SMAD4) (*R* = 0.39, *p* < 0.001), protein secretion pathway (*R* = 0.47, *p* < 0.001), mast cells resting (*R* = 0.21, *p* < 0.001), Type II IFN response (*R* = 0.28, *p* < 0.001) and RPPA INPP4B (*R* = 0.24, *p* < 0.001). In Malignant 4, SLC6A6 showed a significant co‐expression pattern with ELL2 (*R* = 0.45, *p* < 0.001), glycolysis (*R* = 0.46, *p* < 0.001), epithelial mesenchymal transition (*R* = 0.45, *p* < 0.001), Tregs (*R* = 0.39, *p* < 0.001).

CMap analysis was performed with the results of correlation analysis at the pan‐cancer level, and we identified 24 compounds which had been validated to be target‐specific drugs for over 10 cancers including BRCA in multiple clinical trials (Figure 6C). As illustrated in the heat map, most of the predictive inhibitors were negatively correlated with different cancers; inhibitors with negative CMap enrichment scores could reverse the aberrant expression levels of key BRCA cell fate‐related genes, which indicated an antagonistic solid capacity. A set of specific inhibitors targeting representative fate‐related genes from different clusters (puromycin, MS‐275, megestrol, aesculetin), which may reverse the aberrant expression levels of malignant cell fate‐related genes, were extracted for further animal experiments and clinical validation. These results highlighted that the identified inhibitors effectively counteracted the dysregulated expression of key BRCA cell fate‐related genes, reinforcing their potential as therapeutic agents for modulating tumour cell differentiation and progression. We further explored the accessible chromatin landscape (or peak calling) representing potential regulatory regions, likely implicated in the mechanisms underlying key cell fate‐related genes‐driven BRCA bone metastasis, using the ATAC‐seq profile of BRCA patients' biopsies in the TCGA cohort. Importantly, over the whole genome across samples, eight key BRCA cell fate‐related genes were all in the presence of substantial changes in chromatin accessibility widely presenting (Figure 6D).

Results of function enrichment indicated that, in each regulatory network, KEGG pathways where key DEMFRGs most enriched were antigen binding (Malignant 1), axon guidance (Malignant 2) and Rap signalling pathway (Malignant 3), regulation of epithelial cell differentiation, respectively (Figure 6E).

## 4 Discussion

Breast cancer (BRCA) is the leading cancer that affects women worldwide, the incidence rate of which is increasing clearly recently [43]. Despite extraordinary progress in early diagnosis and multi‐modality treatments, the death rate of BRCA is still increasing obviously, remaining a global burden [44, 45]. As lineage differentiation hierarchy and intertumour heterogeneity play an increasingly critical role in cancer, the need for precise molecular subtyping based on cell fate‐determinant genes as prognostic biomarkers remains largely unexplored in bone metastatic BRCA. This gap presents a significant challenge for developing targeted and individualised precision therapies. These unresolved issues impelled us to explore the role of key fate‐related genes in cellular differentiation trajectories and the precise classification of patients with bone metastatic BRCA.

The present computational methods for predicting prognosis of BRCA were mainly based on bulk RNA‐seq data that may result in the following problems. First, the proportions of tumour cells (known as tumour purities) vary in bulk RNA‐seq data among different samples, which may bias the results [46]. Traditional RNA‐seq technology evaluated the average gene expression levels for populations of cells from tumour samples to acquire the bulk RNA‐seq data. Since solid tumour tissues consist of both normal and malignant cells with varying degrees of heterogeneity, bulk RNA‐seq data inherently captures a mixture of signals. The presence of benign components may introduce confounding effects, potentially skewing genomic analyses and leading to biased results [46]. Second, only with bulk RNA‐seq data, it is difficult to figure out how tumour biomarkers are implicated in cellular level perturbation in cancer progression and metastasis. As a new developing computational method, scRNA‐seq has multiple advantages over bulk RNA‐seq. First, scRNA‐seq avoids the tumour purity problem to a certain extent, because it is able to identify the presence of the microenvironment cell clusters from scRNA‐seq data [47]. Second, scRNA‐seq measures the gene expression patterns of each single cell, which can comprehensively elaborate on the cellular perturbations or stages in tumour tissues [48]. In addition, trajectory methods of scRNA‐seq provide a meticulous understanding of dynamic cellular differentiation fate [49, 50]. Moreover, the spatially barcoded arrays allowed unbiased transcriptome profiling in tissue, which can maintain the spatial localisations of sequenced molecules. Therefore, the spatial transcriptome sequence combined with time series of bone metastatic BRCA can not only decode the different cell subpopulations at the single‐cell level, but also achieve comprehensive and accurate analysis concerning the spatial morphological information of bone metastatic BRCA. In the present study, the correlation between cell types, especially the malignant, stromal and immune cells in lesion site, the spatial distributions of which were revealed based on the scRNA‐seq and spatial transcriptome data.

In this study, we systematically analysed the scRNA‐seq and spatial transcriptome data of bone metastatic BRCA patients as well as primary BRCA patients. Here, a total of 4325 scRNA‐seq data were used for UMAP analysis, with 9 clusters and 3 spot types identified where 2493 malignant spots were further extracted utilising canonical marker genes (Malignant1‐3). Extensive intercellular communication was revealed by the iTALK analysis, which suggested that malignant spots occupied the communication centre of the whole duration among BRCA spots. Either through secreted factors or direct cell–cell communication, BRCA malignant spots served as critical and active modulators of tumour and peritumour microenvironment, affecting BRCA bone metastasis. Considering the diversity with a tumour driven by the comprehensive effects of various factors, especially the cell fate‐related genes, activating the intratumor cells and increasing the complexity of the corresponding cell state regulation network, a novel cell‐developmental trajectory was constructed through Monocle 2 in this study. Based on the pseudo‐time analysis, we found that the significant signatures of BRCA intra‐tumoral heterogeneity were mapped to 11 cellular differential states, and the Malignant 2 cluster was located at the starting states of the pseudo‐time trajectory in BRCA, while spots in Malignant 3 and Malignant 1 clusters were located at the terminal states. Additionally, both malignant spot subgroups exhibited the most extensive intercellular communication with others. These results demonstrated that as a dynamic mechanism, cellular communication may be an important factor in determining cell fate and plasticity by the complex action of multiple factors. PCA BRCA score of each sample was calculated based on 34 DEMFRG expression patterns. Patients were stratified into low‐ and high‐PCA BRCA score groups based on the median PCA BRCA score. The prognosis between these two groups differed significantly, and ssGSEA analysis revealed notable differences in immune cell infiltration between them. By analysing the relevant data in the TCIA database, we identified that patients with high‐PCA BRCA score had higher sensitivity to PD‐1 inhibitors and PD‐1 inhibitors in combination with CTLA4 inhibitors. For patients in the low‐PCA BRCA score group, CTLA4 inhibitors alone or no immune checkpoint inhibitors could yield better clinical outcomes. A recent study showed dual inhibition of PD‐L1 and CTLA‐4 contributed to cancer growth arrest and completely blocked distant metastasis, while inhibition of PD‐L1 and CTLA‐4 alone modestly reduced the metastatic spread of malignant cells [51]. Hence, we conclude that for patients with high‐PCA BRCA score, dual immune checkpoint suppressive treatment for PD‐L1 and CTLA‐4 might be the preferred immunotherapy. Univariate, multivariate and Lasso Cox regression analyses identified the 15 most robust prognostic DEMRGs to establish the risk‐related DEMFRG signature model.

Moreover, we constructed a novel BRCA stratification model using a prognostic malignant cell fate‐related gene set. The resulting clustering labels according to differentiation states recapitulated that (1) high maturity malignant cell infiltrating BRCA (HMMCIB), (2) medium‐high maturity malignant cell infiltrating BRCA (MHMMCIB), (3) medium‐low maturity malignant cell infiltrating BRCA (MLMMCIB), (4) low maturity malignant cell infiltrating BRCA (LMMCIB). There were significant differences in the response of the four molecular subtypes to treatment, and the prognosis became worse in turn.

To identify key markers for each subtype, the relative expression levels of prognostic DEMFRGs across the four molecular subtypes were analysed. Notably, the identified marker genes ([HMMCIB, SLIT3], [MHMMCIB, ITGAX], [MLMMCIB, PKIB] and [LMMCIB, MCAM]) were among the core regulatory network genes, suggesting their potentially significant roles in BRCA bone metastasis across different subtypes. These findings will be further explored in the following discussion. In this study, we propose malignant cell maturity‐based clustering as an alternative classification strategy for BRCA. This approach highlights the crucial role of humoral immunity in controlling BRCA metastasis and provides valuable insights for prognosis prediction in bone metastatic BRCA patients. Ultimately, these findings may contribute to advancing individualised precision therapy.

SLIT3 belonged to SLIT proteins that were highly conserved secreted glycoproteins and the ligands for roundabout receptors (ROBOs) [52]. The SLIT/ROBO pathway played a significant role in cellular migration, cellular motility, axon guidance and angiogenesis. Recent research reported SLIT proteins were implicated in tumorigenesis, tumour progression and distant metastasis [53]. SLIT3 had tumour suppressor activity, which was frequently methylated and inactivated in BRCA [54], as well as in cervical cancer [55], lung cancer [54] and colorectal cancer [56]. In breast cancer cell lines, hypermethylation caused silenced SLIT3, which was re‐activated after treating with 5‐aza‐2′‐deoxycytidine Moreover, re‐activation of SLIT3 inhibited the growth and migration of breast cancer cell lines; thereby, it appeared to function as a novel cancer suppressor gene [56]. In our work, SLIT3 was downregulated early and gradually upregulated late along the pseudo‐time, which was highly expressed in HMMCIB subtype with the best prognosis. SLIT3 was highly correlated with signalling pathways related to cell death and apoptosis, including unfolded protein response [57] and inflammatory response [58]. Therefore, our findings suggested that SLIT3 was a master regulator acting at the early and late stages of malignant cell differentiation, which showed pseudotemporal kinetics and was a promising target for BRCA therapy.

Integrins (ITGs) were transmembrane receptors consisting of a cytoplasmic domain implicated in cellular signalling transition, as well as an extracellular domain implicated in ligand binding and recognition [59]. Upon ligand binding, ITGs triggered intracellular signalling pathways which regulated multiple cell functions, such as differentiation, survival, growth and cytoskeleton organisation [60]. They can bind to extracellular matrix (ECM) proteins, including laminin, collagen and fibronectin, ligands on cell surfaces, including intercellular adhesion molecules (ICAMs), as well as soluble ligands, including transforming growth factor‐β (TGF‐β) and fibrinogen [59, 61]. As a member of ITGs, ITGAX was identified to be implicated in the activation of malignant cell proliferation, differentiation, dormancy, survival and metabolic adaptation, as well as in promoting epithelial‐to‐mesenchymal transition (EMT), invasion and distant metastasis [61]. Expression of ITGAX was altered in BRCA and correlated with cancer progression and poor survival [62], which was a novel focus of therapeutic intervention in BRCA. Additionally, ITGAX was found to regulate the expression of programmed cell death protein ligand‐1 (PD‐L1), which was a critical component of the cancer immune evasion machinery, being a promising immunotherapy target clinically effective [63]. In this study, ITGAX was downregulated primarily and gradually upregulated late along the cell‐developmental trajectory, which was highly expressed in MHMMCIB subtype. As a significant regulator of the transition from the immature state to the mature state, ITGAX was also correlated with BRCA bone metastasis, seizing the central role in the bone metastasis‐specific regulatory network of Malignant 1 cell type. Understanding the role of ITGAX and its relationship with immune checkpoints and immune cell infiltration would be an interesting area of focus in BRCA immunotherapy.

The cyclic AMP (cAMP)‐dependent protein kinase inhibitor A (PKA) was a critical regulator with various physiological or pathological effects induced by cAMP. When coupled with G proteins, a variety of ligand–receptor signalling pathways were activated by PKA, controlling cellular growth and determining the direction of differentiation [64, 65]. In patients with BRCA, resistance to therapy was strongly attributed to activated PKA signalling. For instance, in BRCA patients, resistance to endocrine therapy with tamoxifen, the most widely utilised agent with satisfactory efficacy, was significantly related to abnormally high PKA activity [66]. Additionally, activation of PKA pathways was validated to induce chemotherapy resistance in HER2‐positive BRCA patients treated with trastuzumab [67]. Importantly, among three recognised PKA inhibitors (PKI), which included PKI‐α, PKI‐β and PKI‐γ, PKI‐α, also known as PKIA, was the totally specific PKA inhibitor, the suppressing effects of which had been characterised extensively [68, 69]. PKI‐β, known as PKIB, shared just a 40% amino acid identity with PKIA, and the suppressing effects of PKIB on PKA had not yet been fully determined. These regulatory subunits of PKA were aberrantly expressed in BRCA and modified the activity of the PKA pathway [70, 71]. Interestingly, strong cancer grading efficacy of PKIB was also proposed in prostate cancer. More specifically, overexpression of PKIB contributed to castration resistance in prostate cancer through Akt phosphorylation, which was correlated strongly with Gleason grade [72]. Hence, overexpression of PKIB was indicative of a poor prognosis and malignant phenotype in patients with prostate cancer. Further, PKIB may be implicated in changes to the Akt pathway via the phosphorylation of Akt (pAkt), which played an important part in the determination of malignant phenotypes and in the prediction of BRCA progression [73, 74]. High PKIB expression in the cytoplasm of malignant cells was closely associated with pAkt and the poor prognosis of triple‐negative BRCA. All in all, PKIB overexpression may promote the invasion and metastasis of BRCA via upregulating the Akt signalling pathway; in contrast, it may improve chemotherapy resistance by suppressing PKA activity. Therefore, PKIB was considered a novel possible therapeutic target for BRCA, especially in the cancer classification, diagnosis and precise therapy of BRCA. Precise regulation of PKIB was the critical part of target treatment.

Notably, LMMCIB had higher naive malignant cell infiltration and activation of protumour pathways, including hypoxia, EMT and WNT pathways compared with other BRCA subtypes. LMMCIB had a lower proportion of immune infiltrates compared with other subtypes, which mainly correlated with lower anticancer immune cell infiltrates, including CD8+ T cells, DCs and NK cells, and higher protumour immune cell infiltrates, including gamma delta T cells. The immune cell infiltration features of LMMCIB prompted the immune escape and cancer progression in LMMCIB, which partially explained the poorer survival in LMMCIB. Results of molecular subtyping suggested that patients within LMMCIB had the worst survival outcomes, inhibiting its key marker (MCAM) and related cofactors may optimise trans‐differentiation therapeutic strategy and improve patients' prognosis.

The 20 proteins of SRY‐associated high‐mobility group (HMG) box family were a cluster of conserved TFs subdivided into 9 groups, which were characterised by a similar amino acid sequence to the HMG domain [75]. Accumulating studies demonstrated that SOX family proteins played critical roles as regulators in the pathogenesis, invasion and metastasis of cancers [76]. Importantly, the SOXF proteins, namely SOX7, SOX17 and SOX18, were validated to play important roles in cell fate determination and multiple cancer types [77]. SOX17 directed the development and specification of the primitive germ cells, primitive endoderm, definitive endoderm and was therefore implicated in several endoderm‐derived organs and the cardiovascular system.

MCAM, also known as MUC18 or CD146, was firstly observed in malignant melanoma, which had been extensively involved in numerous oncogenic signalling pathways, including VEGF/VEGFR [78], PI3K/AKT [79] and NF‐κB [80], and played as an important driver of progression and metastasis in various cancers, including BRCA [81], lung cancers [82] and melanoma [83]. A recent study reported that MCAM was highly expressed in triple negative BRCA and functioned as a specific EMT activator [84]. Conversely, a study indicated that downregulation of MCAM significantly suppressed EMT activity, leading to obviously weakened tumour growth and distant metastasis of BRCA [85]. Further quantitative proteomic analysis suggested that MCAM‐triggered EMT processes in BRCA cells were induced by negative regulation of ERα [86]. Concerning the therapeutic efficacy of tamoxifen was generally induced by its binding to ER, and the status of ERα had long been proposed as the primary determinant of treatment responses to tamoxifen, therefore, loss of ER expression may cause resistance to chemotherapy [87]. On the other hand, a recent study implied that MCAM can also induce tamoxifen‐based treatment resistance via activating the AKT pathway [81]. Hence, MCAM may serve as a specific predictive biomarker of tamoxifen resistance for BRCA. Clinically, emerging studies consistently indicated MCAM conferred a poor prognosis in BRCA patients [88, 89]. Moreover, upregulation of MCAM was significantly related to poor progression free survival (RFS) and distant metastasis free survival (DMFS) in a large BRCA cohort containing 4142 patients [81]. In particular, overexpression of MCAM was closely related to poor OS, RFS and DMFS in a cohort of ER (+) BRCA patients. More importantly, acting as an adhesion receptor, MCAM was highly expressed by endothelia and smooth muscle cells in blood vessels [90], which was a key component of the endothelial junction [91]. Plasma cell tethering and rolling on the endothelial cells corresponded to the very first steps of the extravasation processes, followed by the adhesion and trans‐endothelial migration of plasma cells [92]. The primary steps engaged plasma cell microvilli at endothelial contact zone and were generally regulated by selectins and ligands of them [93]. MCAM can promote microvilli induction, leading to the slowing of plasma cells in the bloodstream and their rolling on the endothelium surface as well as downstream trans‐endothelial migration [94]. Taken together, MCAM overexpression may inhibit plasma cell infiltration in cancer tissues, weakening antitumour immune response.

In our study, expression level of SOX17 was positively associated with MCAM expression, and the BRCA patients with SOX18/MCAM co‐expression yielded worse prognosis compared to those not co‐expressing aforementioned proteins. These findings indicated that SOX17 was implicated in the invasion and metastasis of BRCA via transactivating its downstream effectors, MCAM. Interestingly, a recent study reported that MCAM was transcriptionally activated by SOX18, a subset of SOX17 interactome, which was indispensable in cancer metastasis [95]. Additionally, resume of MCAM expression reversed the inhibitory effects of SOX18‐downregulation on cancer metastasis, whereas downregulation of MCAM abrogated the enhanced metastatic capacity in SOX18‐overexpressing cancer cells [95]. Here, based on CHIP‐seq and ATAC‐seq analysis, multiple binding peaks of SOX17 were identified in the chromosomal position of MCAM, further validating the binding mechanisms. Using integrated spatial transcriptome and multiomics analyses, we identified that differential expression characteristics of SOX17 and MCAM between UDB subtype (the highest) and other highly differentiated subtypes (relatively lower), and SOX17/MCAM expression was also significantly higher in metastatic BRCA patients than in nonmetastatic BRCA patients. Therefore, we proposed that SOX17/MCAM may be a positive feedback circuit in the metastasis of BRCA, which can deepen our understanding of the progression of BRCA and targeting this signalling axis may be a promising trans‐differentiation therapeutic strategy for the inhibition of BRCA metastasis.

In summary, regardless of current technological advances, the successful development of novel drugs for metastatic BRCA did not always lead to a satisfactory result at the clinical level. Future works of differentiation induction in BRCA should thereby try to focus on: (A) MS‐275 and novel drugs with more predictable effects on malignant cell differentiation and apoptosis, (B) BRCA subtypes that are likely to benefit from differentiation and apoptosis induction using MS‐275, and (C) cytotoxic agents that could be utilised in combination with differentiation induction treatment. Evaluation of the efficacy and safety of unique inhibitors will also be our future research directions.

However, our study has certain limitations. Firstly, the resolution constraints of spatial transcriptomics may limit the full characterisation of BRCA tumour heterogeneity. Additionally, as our findings are primarily based on bioinformatics analysis, our explorations were not able to fully recapitulate the developmental trajectories and the diversities of cellular differentiation states within BRCA malignant cells. Furthermore, the inferred developmental trajectories and drug screening results lack multi‐dimensional validation, more cellular and animal experiments, as well as clinical trials, will be necessary to confirm the therapeutic efficacy of the identified inhibitors and validate their clinical relevance in future. Despite these limitations, our study provides key insights into bone metastatic BRCA, revealing developmental stage‐based therapeutic vulnerabilities and potential diagnostic and prognostic biomarkers, laying a foundation for future translational research.

## Author Contributions

**Penghui Yan:** conceptualization (lead), data curation (supporting), formal analysis (supporting), funding acquisition (equal), investigation (lead), resources (supporting), software (supporting), supervision (lead), validation (lead), visualization (lead), writing – original draft (lead), writing – review and editing (lead). **Hanlin Sun:** conceptualization (supporting), data curation (lead), formal analysis (supporting), investigation (equal), methodology (equal), project administration (equal), software (supporting), supervision (supporting), visualization (supporting), writing – original draft (equal). **Siqiao Wang:** data curation (equal), investigation (supporting), methodology (supporting), software (equal), supervision (equal), validation (equal), visualization (supporting), writing – original draft (equal). **Runzhi Huang:** writing – original draft (equal). **Chaofeng Shi:** validation (supporting), visualization (supporting), writing – original draft (supporting). **Qihang Yang:** investigation (supporting), methodology (supporting), project administration (supporting), writing – original draft (supporting). **Yibo Qiao:** data curation (supporting), formal analysis (supporting), writing – original draft (supporting). **Haonan Wang:** formal analysis (supporting), supervision (supporting), validation (supporting), visualization (supporting), writing – original draft (equal). **Deqian Kong:** data curation (supporting), software (supporting), supervision (supporting), writing – original draft (equal). **Jiwen Zhu:** methodology (supporting), project administration (supporting), writing – original draft (supporting). **Yunqing Yang:** methodology (lead), validation (lead), writing – original draft (equal), writing – review and editing (lead). **Zongqiang Huang:** conceptualization (equal), funding acquisition (equal), methodology (lead), validation (lead), writing – review and editing (lead).

## Ethics Statement

The study was approved by the Ethics Committee of the Shanghai Tongji Hospital affiliated to Tongji University.

## Consent

The authors have nothing to report.

## Conflicts of Interest

The authors declare no conflicts of interest.

## Supporting information


Data S1.

## Data Availability

The data sets generated and/or analysed during the current study are available in the GEO (https://www.ncbi.nlm.nih.gov/geo/) database (GSE113197), The Cancer Genome Atlas (TCGA) database (https://tcga‐data.nci.nih.gov), Cistrome Cancer database (http://cistrome.org/) and ImmPort database (https://www.import.org/).
