# Supplementary material for: Molecular Subtypes and Risk Prediction Model Based on Malignant Cell Differentiation Trajectories in Breast Cancer
Source: J Cell Mol Med. 2025 Aug 8;29(15):e70680. doi: 10.1111/jcmm.70680 (PMC12332891; doi:10.1111/jcmm.70680)
Supplement: Supplementary file 1 — Data S1. [file JCMM-29-e70680-s001.docx]

**Supplementary materials**

**
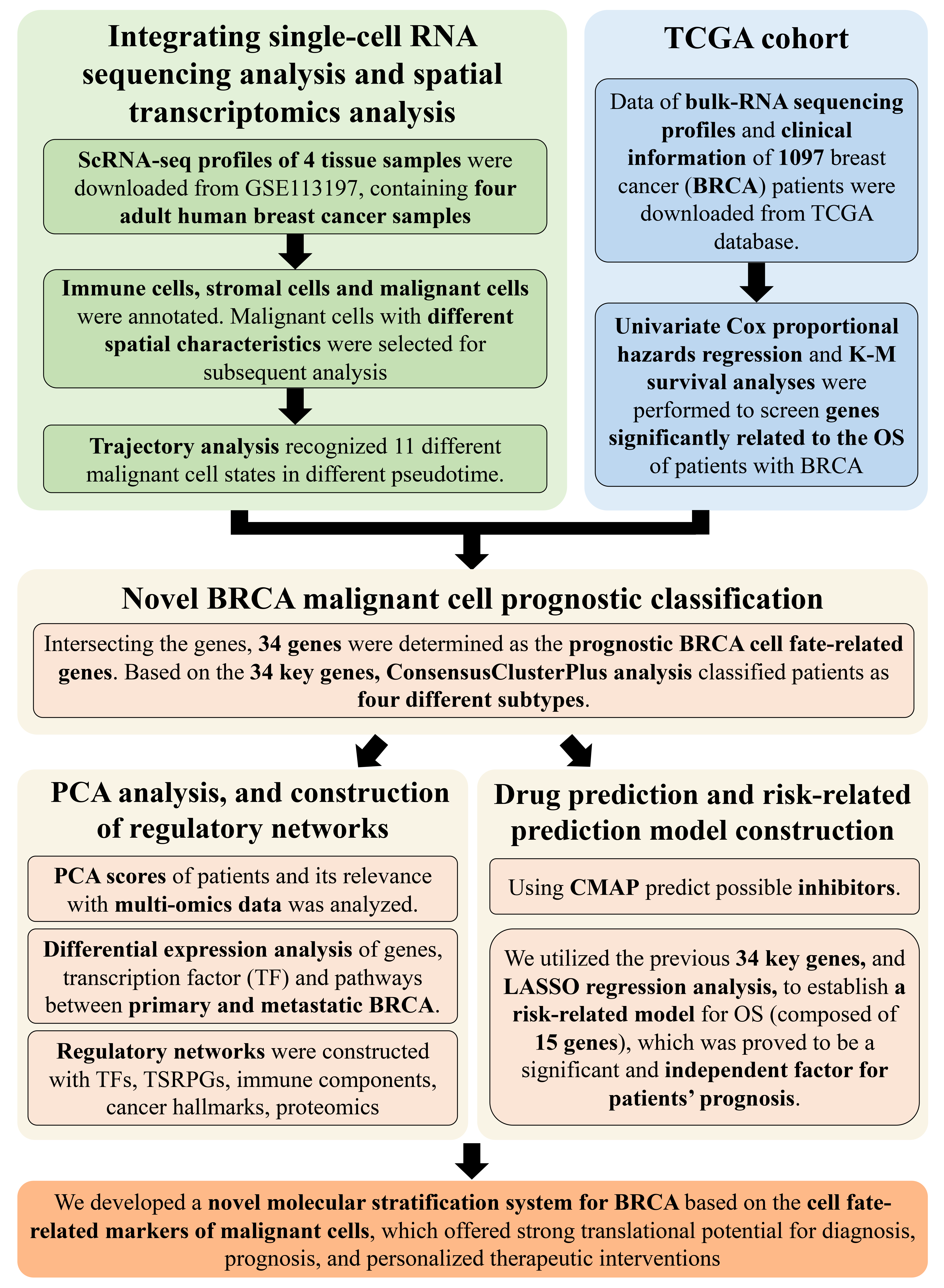
**

**Figure S1: The detailed flowchart of this study was presented in Figure S1, illustrating the primary processes of our study.**

**
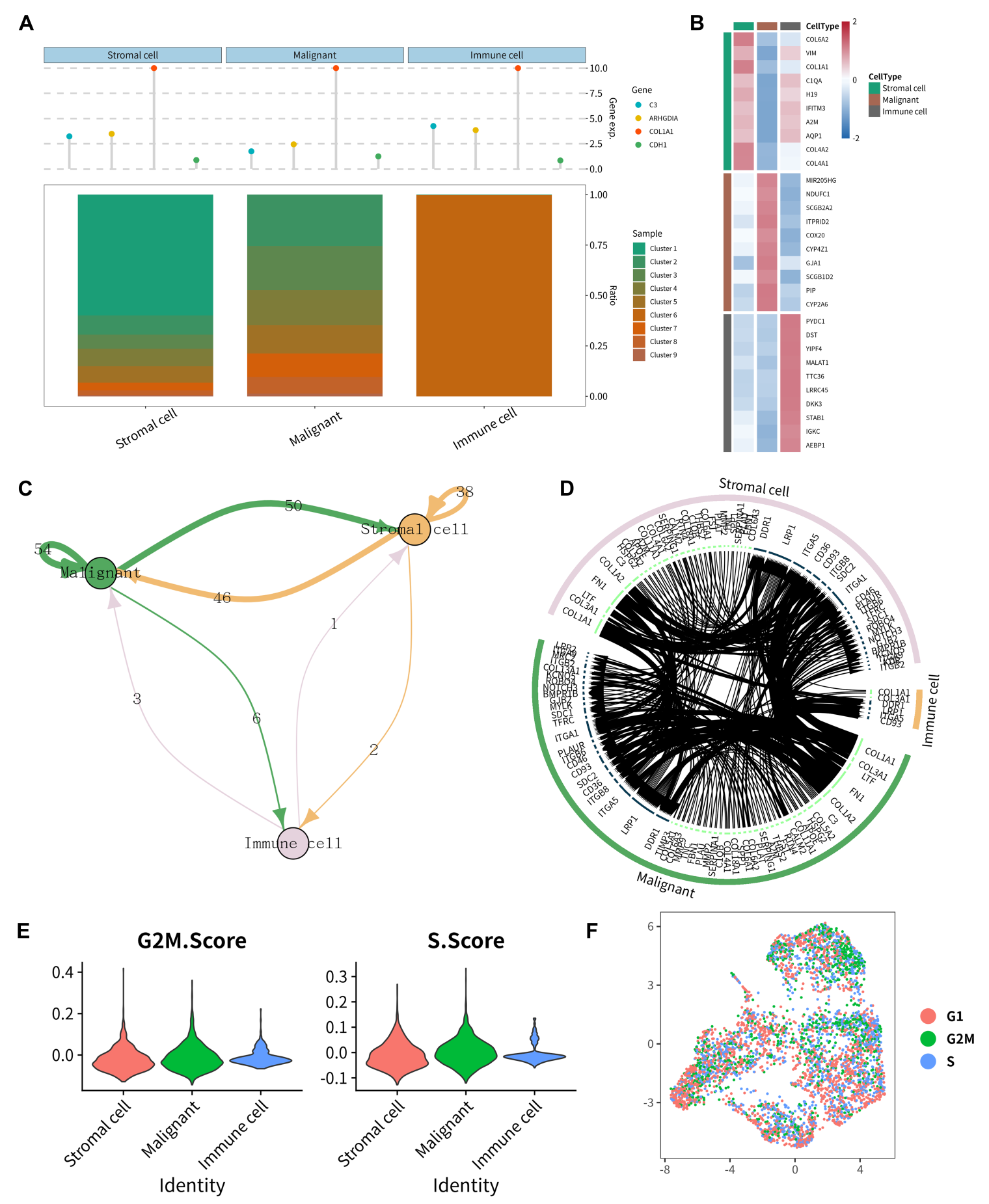
**

**Figure S2: Integrated analysis of differential expression gene (DEG), cellular communication, and cell cycle for all cell clusters**

1. Cleveland dot plot showed the expression levels of classical marker genes in different subpopulations and Stacked bar plot showed the distribution of cell types.
2. Co-expression correlation heat map showed the expression levels of the top 10 DEGs of each cell type.
3. UMAP iTALK network showing the intersected cellular communication among 3 main cell clusters, with different subpopulations color-coded.
4. UMAP ligand-receptor plot showing the connection degree of 3 main cell clusters, as well as the key genes that were implicated in the cell communication.
5. Violin plots showed the G2M.Score and S.Score of 3 main cell clusters.
6. Feature plot illustrated the distribution of 3 main cell clusters at different cell cycle phases.


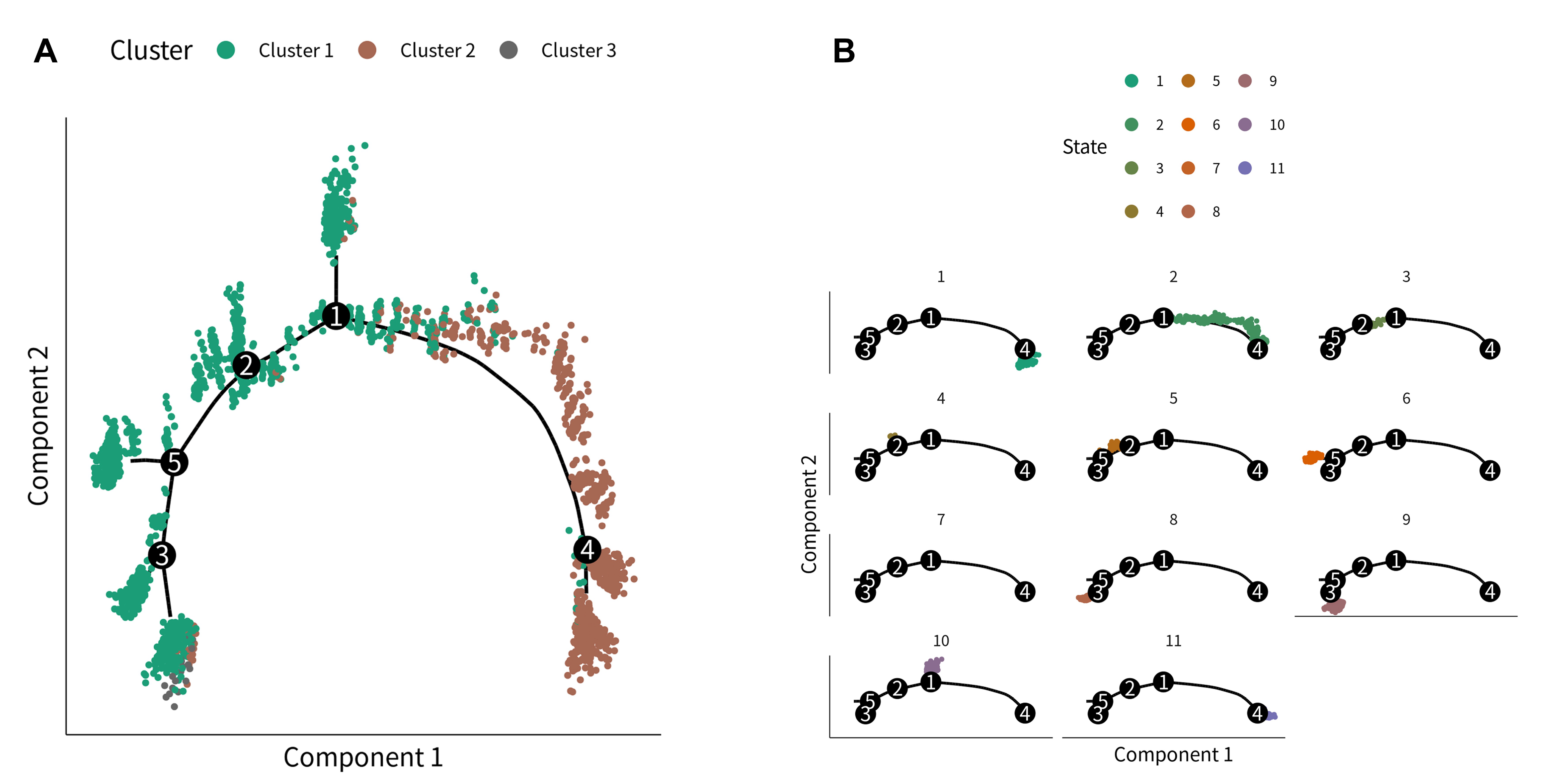


**Figure S3: Single cell trajectory analysis of malignant cells**

1. The subpopulation of malignant cells from BRCA contributing to each state was given in the relevant trajectory, which were labeled with the corresponding cell types.
2. The differentiated fates were separated into 11 cell states by branch points 1 to 5, and individual states were illustrated in the trajectory.
3. Dynamically changed expression levels of representative fate-related genes in malignant cells for distinct differentiation states.
4. Relative expression levels of fate-related genes in each subpopulation calculated by Monocle2.


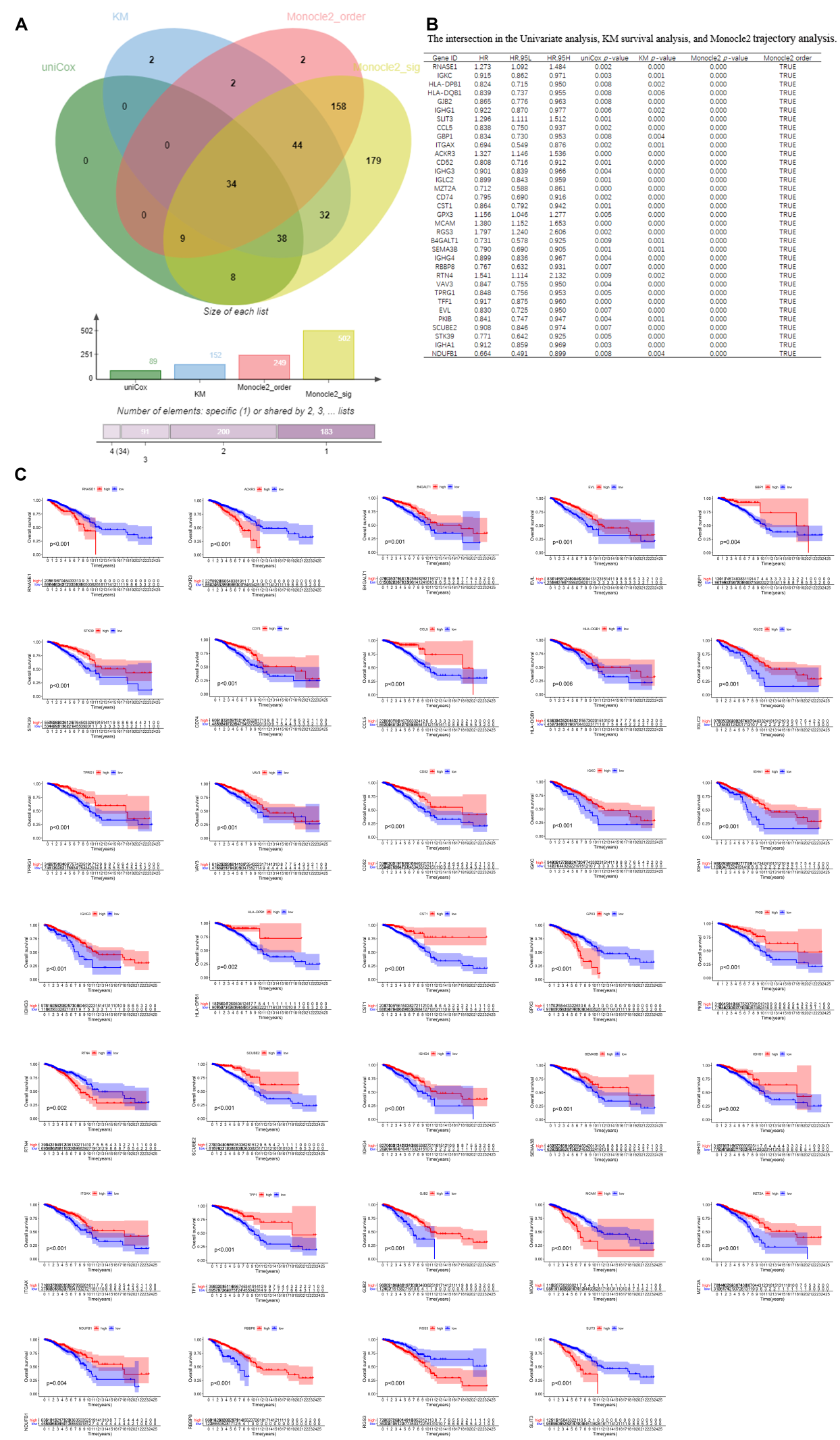


**Figure S4: Identification of malignant cell fate-related gene set in bone-metastatic BRCA**

1. Venn diagram showed genes overlapping of survival analyses and pseudo-time analyses, which were termed prognostic BRCA cell fate-related gene set.
2. Results of univariate Cox (uniCox) regression analysis and Kaplan–Meier (KM) survival analysis of 34 prognostic BRCA cell fate-related genes.
3. KM survival curves showed significant correlation between overall survival and 34 prognostic BRCA cell fate-related genes (all *P* < 0.05).


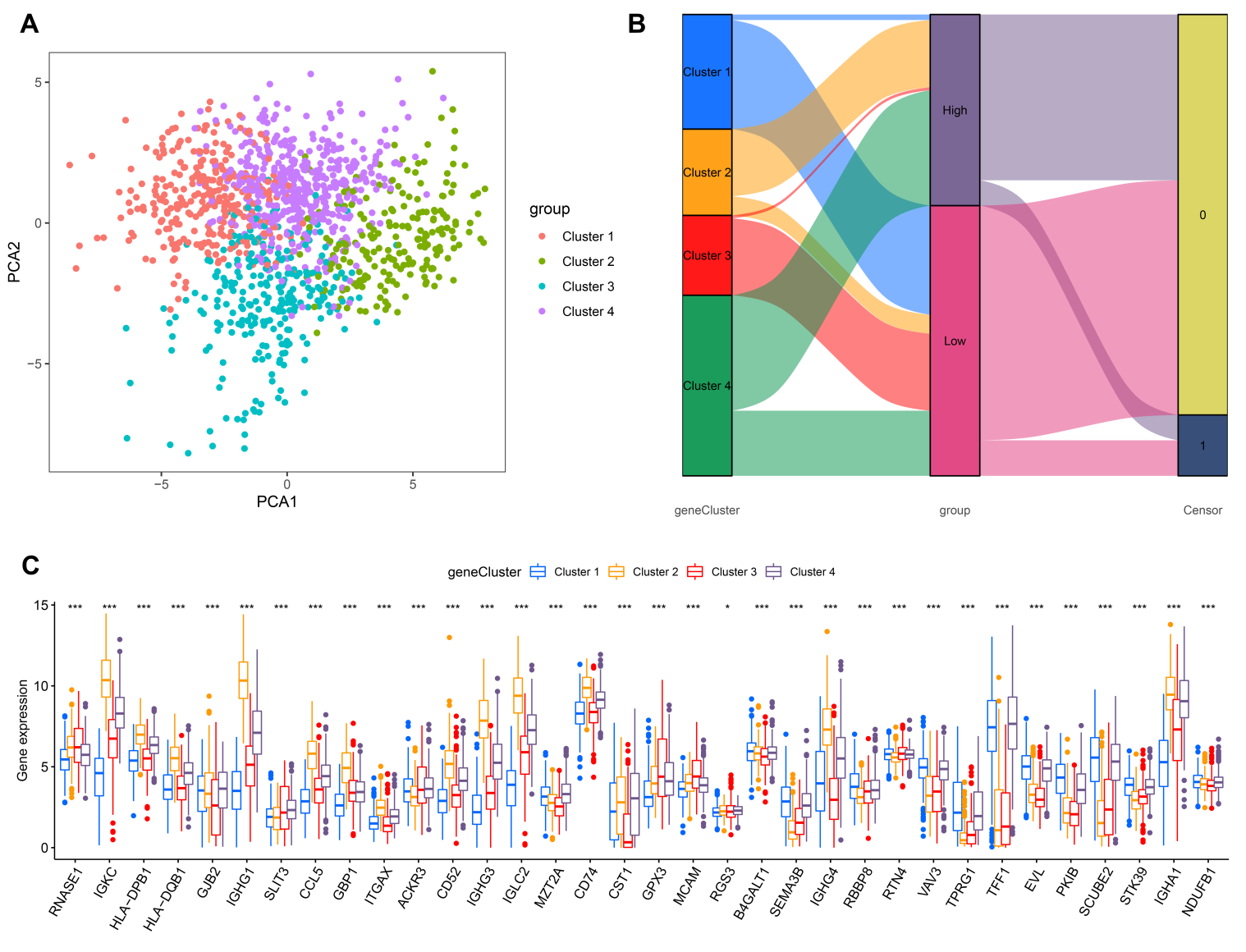


**Figure S5: Using Prognostic BRCA cell fate-related genes for the consensus clustering of BRCA TCGA cohorts**

1. Evaluation of PCA BRCA score for the four clusters by Mann–Whitney U-test.
2. Sankey diagram showed the association between clustering, PCA BRCA score and alive status.
3. Box plot showed significantly differential expression patterns of 34 malignant cell fate-related genes among 4 patient groups (all *P* < 0.05).

**
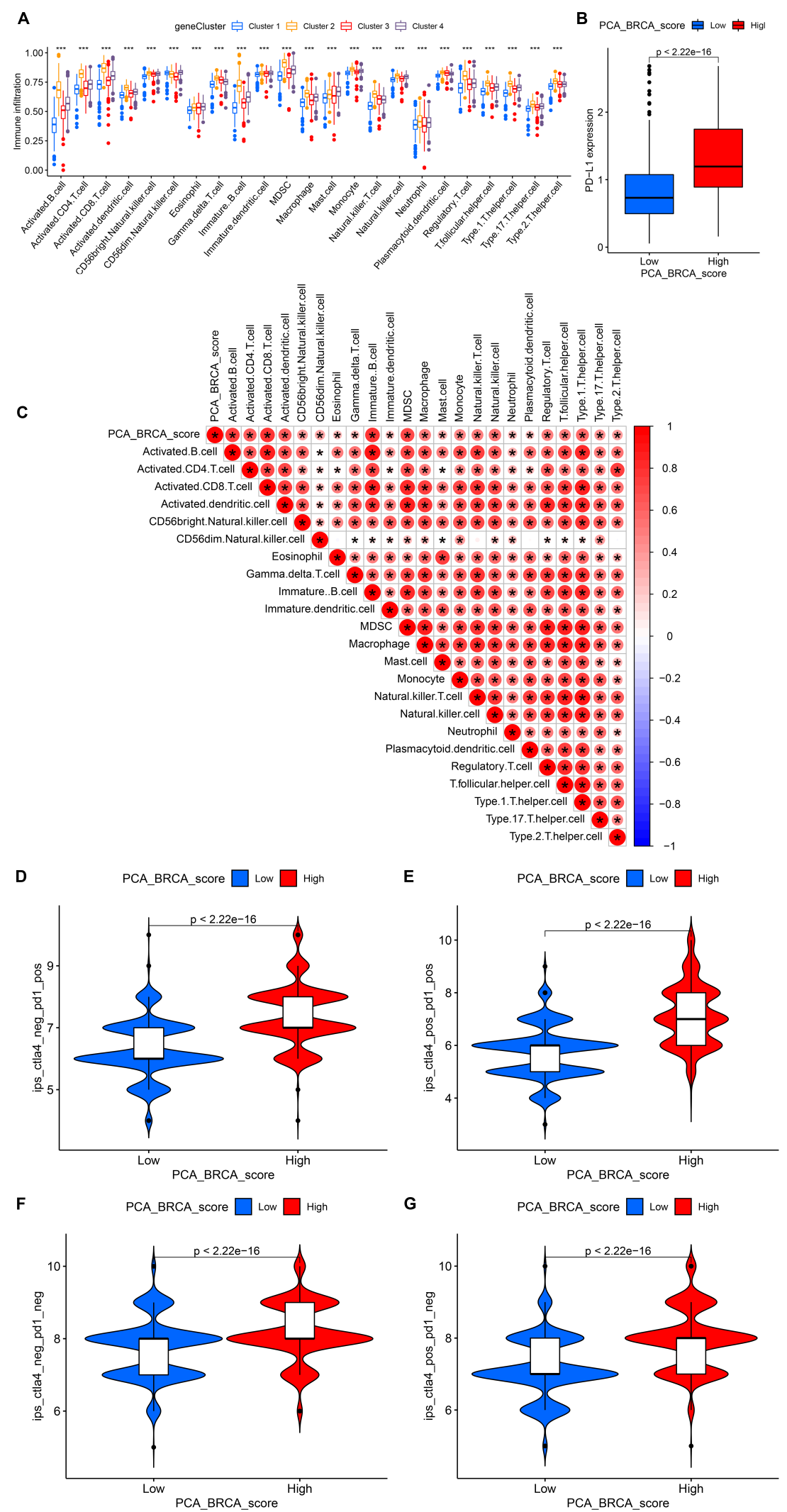
**

**Figure S6: Multi-omics analyses of bulk RNA-seq data in TCGA based on prognostic BRCA cell fate-related gene set (immune infiltration)**

1. Box plot show significantly infiltration degree of immune cells among four Consensus clusters (*P* < 0.05).
2. Significant upregulation of immune checkpoint PD-L1 was observed in high PCA BRCA score group (*P* < 0.001).
3. A correlation matrix of PCA BRCA score and 23 types of immune cells in BRCA, which indicated a strong relationship between them.

**(D-F)** Role of PCA BRCA score in immunotherapy sensitivity.

**
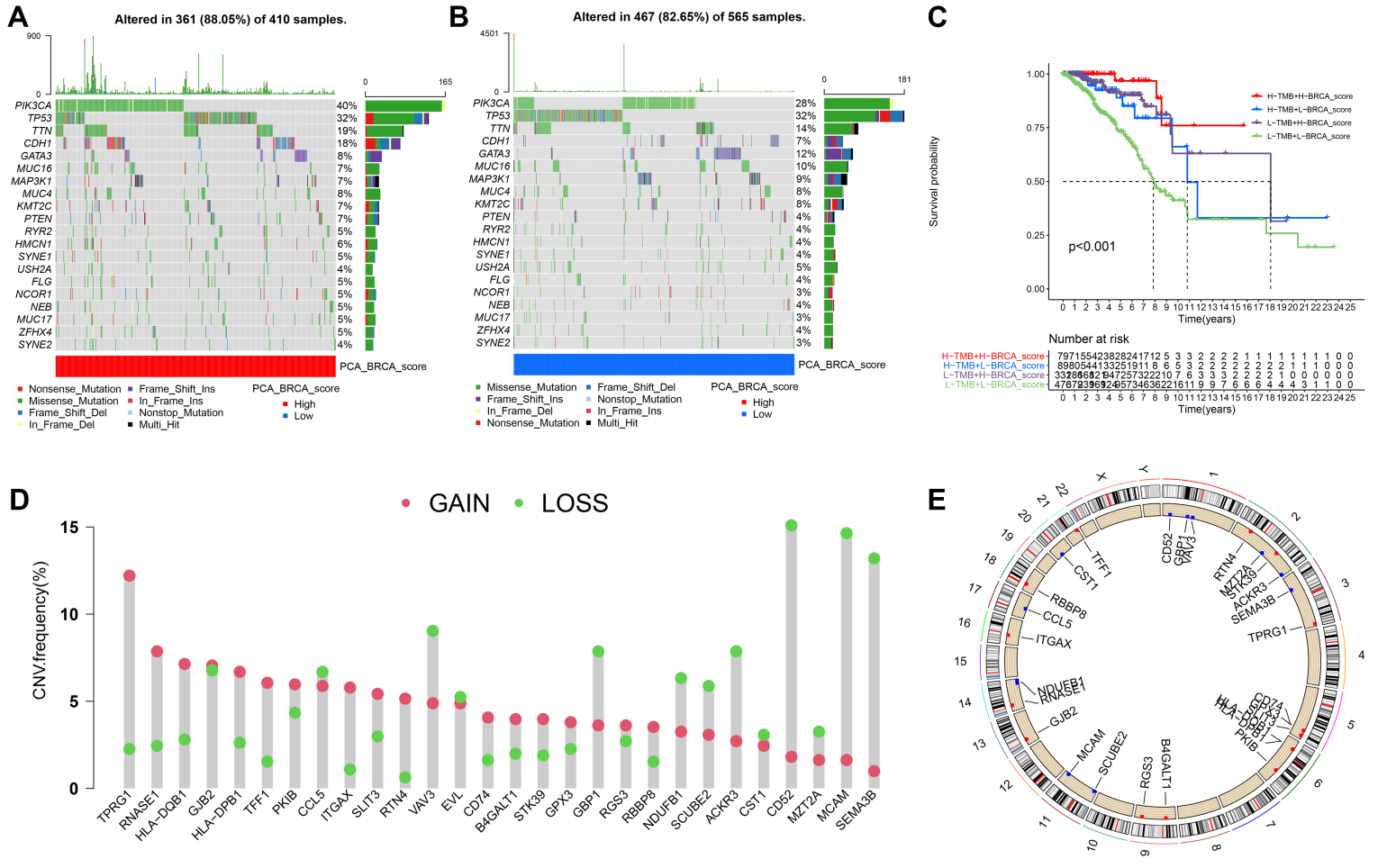
**

**Figure S7: Integrated multi-omics analyses of bulk RNA-seq data in TCGA based on prognostic BRCA cell fate-related gene set (genome characteristics)**

1. Waterfall plots showed the types and frequency of gene mutations in samples from the high PCA BRCA score groups.
2. Waterfall plots showed the types and frequency of gene mutations in samples from the low PCA BRCA score groups.
3. Prognosis analysis of the combination of BRCA subgroups. H-TMB (high tumor mutation burden); L-TMB (low tumor mutation burden); H-BRCA score (high PCA BRCA score); L-BRCA score, (low PCA BRCA score).
4. CNV analysis in 28 prognostic BRCA cell fate-related genes, with copy number gains for red and losses for green.
5. Circus plot was constructed to visualize CNVs inBRCA samples, with copy-number alterations shown at the center and mutations in genes at the outermost ring. Regions marked in red indicated the copy number gain in CNV, while blue points indicated the copy number loss of that region.

**
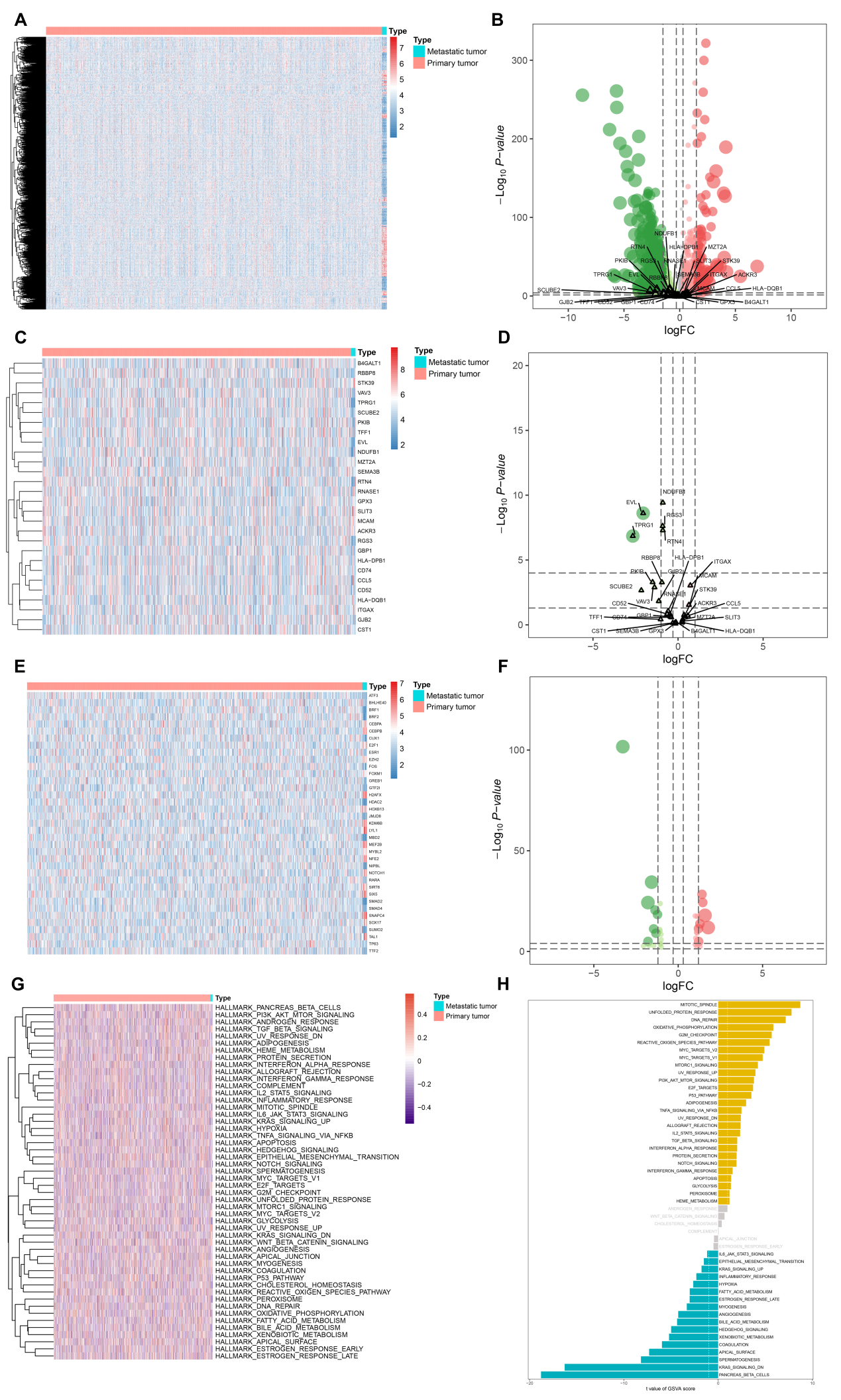
**

**Figure S8: Component analysis of regulation network (differentially expressed genes (DEGs), transcription factors (DETFs), and signaling pathways in BRCA), related to Figure 5**

1. Heat map showed the expression levels of DEGs between metastatic tumor samples and primary tumor samples.
2. Volcano plot showed the differential expression levels of DEGs between bone metastatic tumor samples and primary tumor samples, where red represented up-regulation while green represented down-regulation.
3. Heat map showed differential expression level of 28 cell fate-related genes between bone metastatic tumor samples and primary tumor samples.
4. Volcano plot with specific gene location showed regulation level 28 cell fate-related genes between bone metastatic tumor samples and primary tumor samples.
5. Heat map showed the expression levels of 37DETFs (DETFs) between bone metastatic tumor samples and primary tumor samples.
6. Volcano plot showed the differential expression patterns of these DETFs between bone metastatic tumor samples and primary tumor samples, where red represented up-regulation while green represented down-regulation.
7. Heat map illustrated the differential regulation level of 50 hallmark signaling pathways between bone metastatic tumor samples and primary tumor samples.
8. Bar plot showed the *t* score of signaling pathways by Gene Set Variation Analysis (GSVA). 44 significantly differentially expressed pathways were more intuitively visualized in the histogram.

**
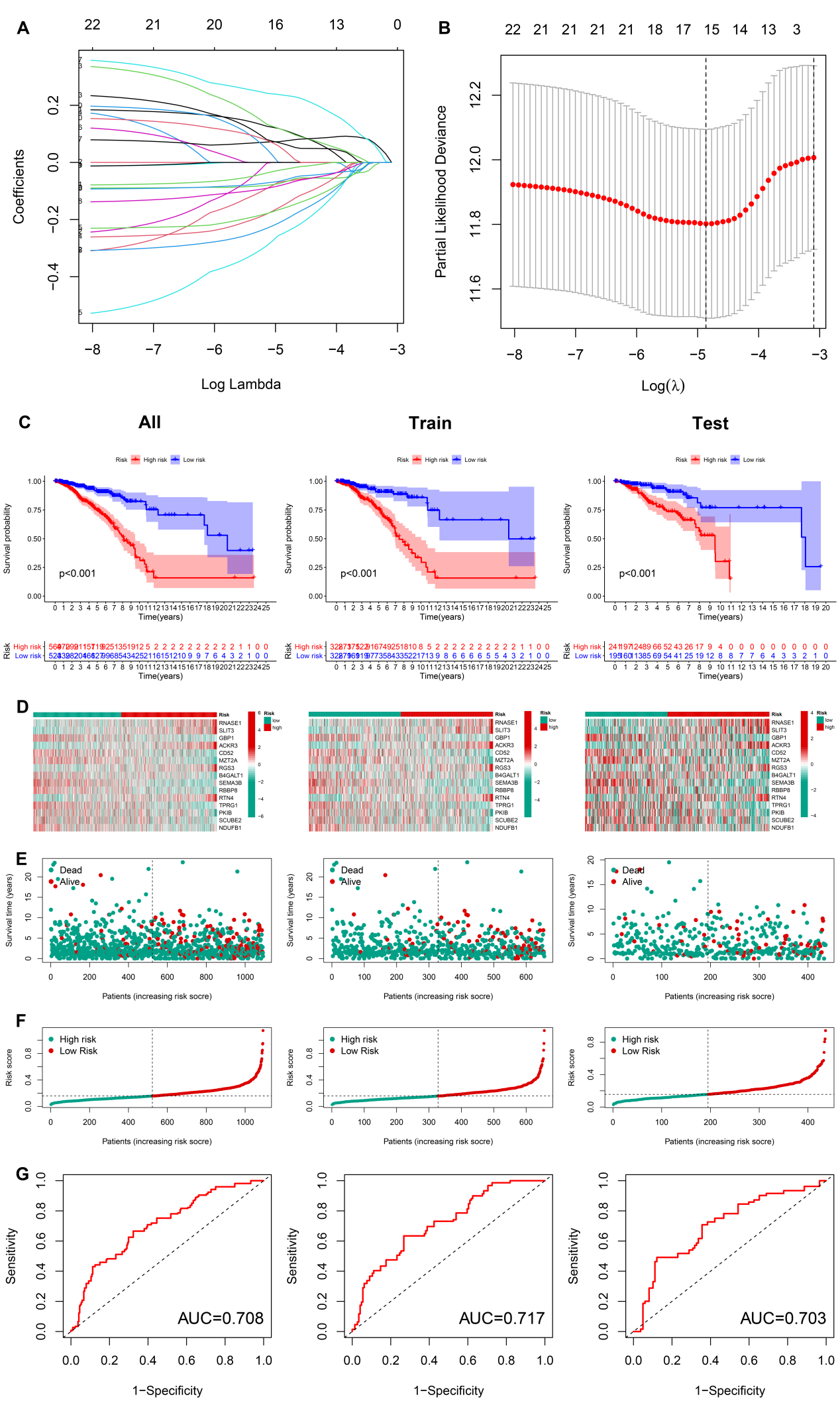
**

**Figure S9: Construction of a risk score model for prediction of prognosis**

1. Coefficients in the Lasso regression for prognostic malignant cell fate-related genes screening.
2. Cross-validation for tuning para-meter identification in the proportional hazards model.
3. Kaplan-Meier (KM) curve showed the low-risk subgroup had a significantly better prognosis than the high-risk subgroup, indicating the broader clinical utility of prognostic BRCA cell fate-related gene signature in all set, train set, and test set, respectively.
4. Heat maps showed the expression level of 15 prognostic malignant cell fate-related genes identified using Lasso regression in all set, train set, and test set, respectively.
5. Distribution of all BRCA patients’ survival status was displayed in the scatter plot in all set, train set, and test set, respectively.
6. Based on the TCGA data profiling, the risk plot showed the risk score distribution for all patients with BRCA, red and green representing high-risk and low-risk subgroups in all set, train set, and test set, respectively.
7. Receiver operating curve (ROC) for evaluating the reliability of the risk prediction model in all set, train set, and test set, respectively.

**
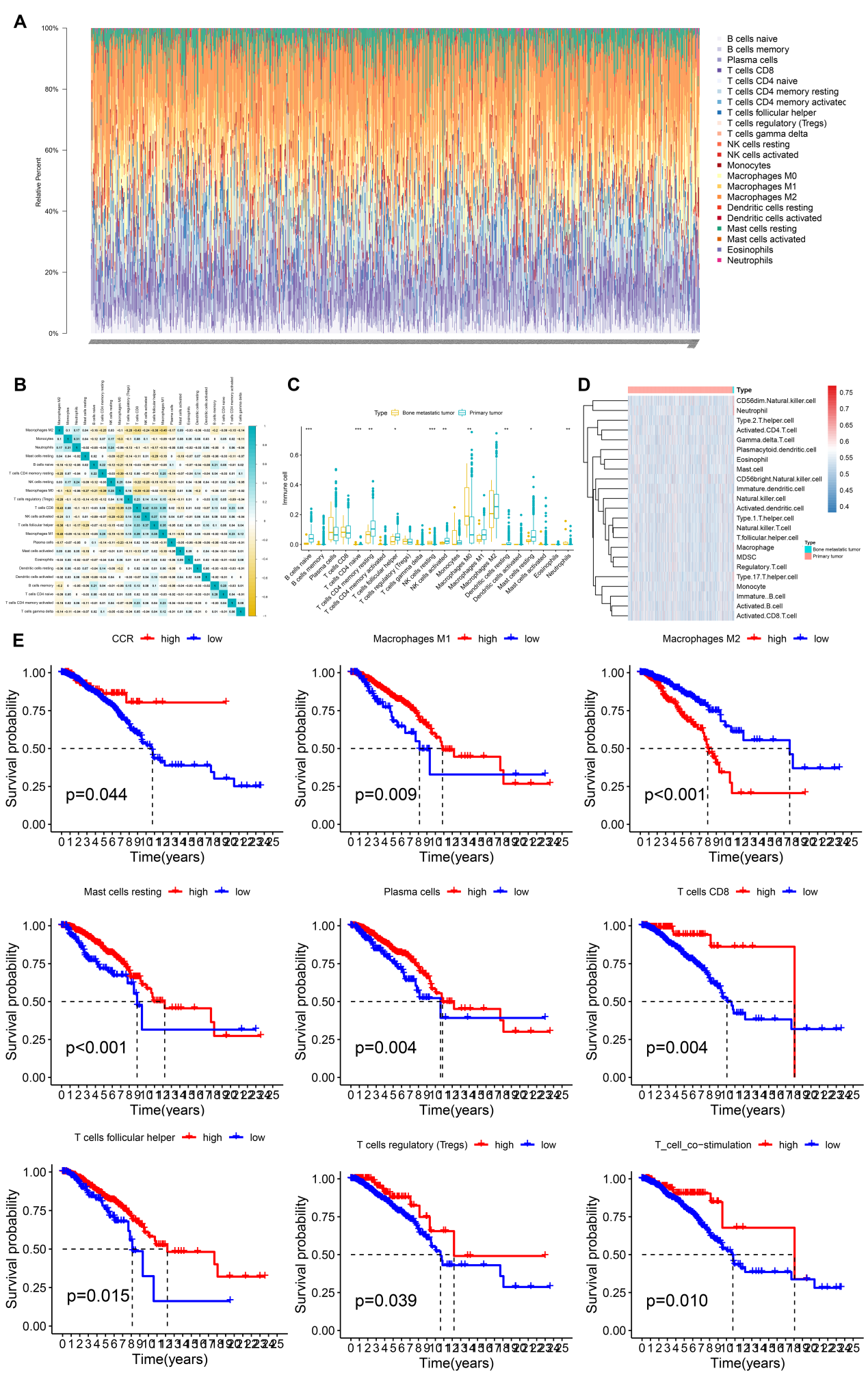
**

**Figure S10: Component analysis of regulation network (immune infiltration profiling of BRCA)**

1. Stacked histogram showing the composition of 22 types of immune cells estimated by CIBERSORT algorithm.
2. Correlation heat map of 22 immune proportions in BRCA.
3. Comparison of the fractions of immune cell subpopulations between primary BRCA samples and bone metastatic BRCA samples.
4. Heat map showed expression level of 22 types of immune cells between primary and bone metastatic BRCA tumor based on the ssGSEA score.
5. Kaplan-Meier (KM) curve showed BRCA patients with high infiltration degree of CCR, macrophages M1, mast cells resting, plasma cells, T cells CD8, T cells follicular helper, T cells regulatory (Tregs) exhibited a better prognosis, whereas those with high infiltration degree of macrophages M2 showed a poorer prognosis (all *P* < 0.05).
